# Fullerol Attenuates Diabetic Muscle Atrophy and Ferroptosis-Associated Injury: Intervention Timing Influences Myogenesis-Associated Responses In Vitro

**DOI:** 10.3390/life16101746

**Published:** 2026-10-17

**Authors:** Zengguang Wang, Yuxin Zhang, Chen Xu, Dongyun Gu

**Affiliations:** 1 Department of Orthopaedic Surgery, Shanghai Ninth People’s Hospital, Shanghai Jiao Tong University School of Medicine, Shanghai 200011, China; 2 School of Biomedical Engineering and Med-X Research Institute, Shanghai Jiao Tong University, Shanghai 200011, China; 3 Department of Oral Surgery, Shanghai Ninth People’s Hospital, Shanghai Jiao Tong University School of Medicine, Shanghai 200011, China

**Keywords:** fullerol, diabetic muscle atrophy, ferroptosis, glucolipotoxicity, myogenesis-associated responses, intervention timing

## Abstract

Glucolipotoxic stress contributes to diabetic muscle atrophy by disrupting myoblast homeostasis and myogenesis-associated responses. Fullerol is a water-soluble polyhydroxylated fullerene derivative with potent free-radical-scavenging activity. We investigated the protective effects of fullerol and compared concurrent and post-HGPA intervention schedules in C2C12 myoblasts. We evaluated C2C12 myoblasts exposed to high glucose plus palmitic acid (HGPA), RAW264.7 macrophages, macrophage-conditioned medium, and male C57BL/6J mice subjected to a high-fat diet (HFD) and streptozotocin (STZ). In the animal experiment, fullerol was administered locally during diabetic model development, whereas intervention timing was compared only in the C2C12 model. In myoblasts, HGPA induced Fe^2+^ and superoxide anion accumulation, lipid peroxidation, GSH depletion, mitochondrial depolarization, increased ACSL4, and decreased GPX4, all of which were attenuated by fullerol. Fullerol also attenuated erastin-induced ferroptosis-associated injury. Concurrent treatment preserved proliferation-associated markers, reduced P21 and γ-H2AX, restored Myod1 and Myog expression and MYHC protein abundance, and suppressed Murf1 and Atrogin-1 expression. At the 48-h endpoint, both concurrent and 24-h post-HGPA fullerol treatment attenuated ferroptosis-associated changes. At the separate 96-h endpoint, post-HGPA fullerol treatment only partially restored proliferation- and myogenesis-associated marker profiles, even when fullerol treatment was continued for 72 h. In macrophages, fullerol reduced CCR7 and TNF-α and increased CD206 and SOD1. Conditioned medium from HGPA-plus-fullerol-treated macrophages induced less superoxide anion and Fe^2+^ accumulation in myoblasts than medium from HGPA-stimulated macrophages. In diabetic mice, fullerol preserved myofiber size and relative muscle weight, improved myogenesis-associated gene and protein expression, suppressed atrophy-associated gene expression, reduced 4-HNE accumulation and F4/80-positive macrophage abundance, and normalized GPX4 and ACSL4 protein expression toward control levels. These findings support the protective potential of fullerol during the development of diabetic muscle atrophy and indicate that intervention timing influences proliferation- and myogenesis-associated responses in vitro.

## 1. Introduction

Diabetic muscle atrophy, a frequently underrecognized yet debilitating complication of diabetes mellitus, significantly impairs quality of life and contributes to reduced muscle strength, impaired mobility, and physical disability in affected individuals [1,2]. The development of diabetic muscle atrophy is multifactorial, involving insulin resistance, metabolic dysfunction, oxidative stress, chronic inflammation, and impaired muscle regeneration. Persistent hyperglycemia and hyperlipidemia generate a glucolipotoxic environment that promotes skeletal muscle injury [3,4,5]. Although diabetic muscle atrophy shares several pathological features with age-related sarcopenia, it develops in the context of sustained metabolic stress that accelerates oxidative injury, metabolic inflammation, and cellular dysfunction [6,7,8,9,10]. Skeletal muscle homeostasis and repair rely on satellite cell activation followed by the proliferation and differentiation of myogenic progenitors [11].

Recent evidence has implicated ferroptosis, an iron-dependent form of regulated cell death driven by lipid peroxidation, in tissue injury associated with metabolic diseases and diabetic complications, including diabetic osteoporosis, retinopathy, and non-alcoholic fatty liver disease [12,13,14]. Emerging studies have also suggested the involvement of iron dysregulation and lipid peroxidation in diabetes-associated skeletal muscle injury; however, direct evidence linking ferroptosis to diabetic muscle atrophy remains limited [15,16]. Prevention of cell death and restoration of cellular function represent biologically distinct outcomes [17].

In addition to directly damaging muscle cells, the diabetic microenvironment is characterized by persistent low-grade inflammation that further impairs muscle regeneration [18,19]. Macrophages are critical regulators of the skeletal muscle regenerative niche, and the balance between pro-inflammatory and pro-regenerative states influences myoblast proliferation and differentiation. Sustained pro-inflammatory macrophage signaling can suppress myogenesis, whereas a shift toward a reparative phenotype may create a paracrine environment favorable for muscle regeneration [20,21]. Thus, an effective intervention may need to protect myoblasts from metabolic injury while also modulating macrophage redox–inflammatory responses.

Fullerol, a polyhydroxylated derivative of fullerenes, has garnered significant attention in nanomedicine due to its potent free-radical-scavenging capabilities and excellent biocompatibility [22,23]. Its carbon cage and multiple hydroxyl groups contribute to its redox-regulating activity [24,25]. The influence of administration timing on these effects and the ability of fullerol to regulate macrophage inflammatory and antioxidant responses have also not been defined. The HFD/STZ mouse model is commonly used to reproduce diet-induced metabolic dysfunction and persistent hyperglycemia associated with type 2 diabetes [26].

In this study, we aimed to determine whether the timing of fullerol administration influences its effects on HGPA-induced myoblast injury in vitro and to evaluate the protective effects of local fullerol administration during the development of diabetes-associated skeletal muscle atrophy in vivo. We compared concurrent and post-HGPA fullerol administration by assessing ferroptosis-associated changes and proliferation- and myogenesis-associated markers in C2C12 myoblasts. We also evaluated macrophage-associated inflammatory and redox responses, macrophage-conditioned-medium effects on myoblasts, and skeletal muscle atrophy-related outcomes in HFD/STZ-treated mice receiving fullerol from the beginning of model induction.

## 2. Materials and Methods

### 2.1. Materials and Reagents

Fullerol was obtained from XFNANO (100778, Nanjing, China), and Palmitic acid was purchased from Beyotime Biotechnology (ST3329, Shanghai, China). Streptozotocin (ST1668, Beyotime), Mannitol (Y265929, Beyotime), Erastin (SC0224, Beyotime), Ferrostatin-1 (SH2738, Beyotime), DAPI (C1006, Beyotime), PBS (C0221A, Beyotime), Liproxstatin-1 (54021ES08, Yeasen, Shanghai, China), Z-VAD-FMK (56679ES10, Yeasen), and Necrostatin-1 (53771ES10, Yeasen) were also acquired. The primary antibodies employed included ACSL4 (22401-1-AP, Proteintech, Wuhan, China), GPX4 (30388-1-AP, Proteintech), P21 (10355-1-AP, Proteintech), γ-H2AX (83307-2-RR, Proteintech), KI67 (27309-1-AP, Proteintech), CCNA2 (18202-1-AP, Proteintech), CD206 (18704-1-AP, Proteintech), CCR7 (25898-1-AP, Proteintech), TNF- α (26162-1-AP, Proteintech), SOD1 (10269-1-AP, Proteintech), GAPDH (60004-1-Ig, Proteintech), MYHC (14-6503-80, Invitrogen, Carlsbad, CA, USA), F4/80 (28463-1-AP, Proteintech), 4-Hydroxynonenal (4-HNE, A26085, Abclonal, Wuhan, China), and Myogenin (A17427, Abclonal). The secondary antibodies employed included CoraLite594-conjugated Goat Anti-Rabbit IgG(H + L) (SA00013-4, Proteintech), CoraLite488-conjugated Goat Anti-Rabbit IgG(H + L) (SA00013-2, Proteintech), HRP-conjugated Goat Anti-Rabbit IgG(H + L) (SA00001-2, Proteintech), and HRP-conjugated Goat Anti-Mouse IgG(H + L) (SA00001-1, Proteintech).

### 2.2. Characterization of Fullerol and Assay-Interference Controls

Fullerol was dispersed in deionized water before characterization. Transmission electron microscopy (TEM; JEM-F200, JEOL Ltd., Tokyo, Japan) was used to characterize the morphology and size of the fullerol nanoparticles. The diameters of individual particles in the TEM images were quantified using ImageJ software (version 1.54p, National Institutes of Health, Bethesda, MD, USA). The hydrodynamic diameter and zeta potential of the nanoparticles were measured using a Zetasizer Nano ZS90 analyzer (Malvern Instruments Ltd., Malvern, UK). To characterize fullerol under the biologically relevant exposure conditions used in the cell experiments, fullerol was further diluted to 20 μg/mL in complete culture medium consisting of DMEM supplemented with 10% fetal bovine serum and 1% penicillin–streptomycin. The hydrodynamic diameter and zeta potential of fullerol in the complete culture medium were measured using the same Zetasizer Nano ZS90 analyzer under identical measurement settings.

Potential interference of fullerol with the absorbance- and fluorescence-based assays was also evaluated. For the CCK-8 interference test, four conditions were examined: CCK-8 reagent alone, CCK-8 reagent supplemented with 20 μg/mL fullerol, cell-generated CCK-8 reaction product, and cell-generated CCK-8 reaction product supplemented with 20 μg/mL fullerol. Absorbance at 450 nm was measured using an Infinite M200 Pro microplate reader (Tecan, Männedorf, Switzerland).

For the fluorescence-interference controls, cells were stained with DHE, RhoNox-6, or JC-1 according to the corresponding assay protocols and then exposed to vehicle or 20 μg/mL fullerol for 2 min immediately before fluorescence imaging. Images within each assay were acquired using identical exposure time, illumination intensity, gain, and magnification. A two-step correction procedure was used for fluorescence quantification. First, the mean fluorescence intensity of a representative cell-free region within each image was subtracted from that of the corresponding cellular region of interest to correct for local image background. Second, the background-corrected cellular fluorescence measured in the corresponding probe-free control images was subtracted from that measured in the probe-stained images to correct for cellular and fullerol-associated autofluorescence. Probe-free controls were prepared under the corresponding vehicle- or fullerol-treated conditions and imaged using the same acquisition settings. For JC-1 analysis, these corrections were performed separately for the red and green channels before calculating the red and green fluorescence intensity. The corrected DHE, RhoNox-6, and JC-1 fluorescence readouts were subsequently compared between the vehicle- and fullerol-treated conditions.

### 2.3. Cell Culture

Murine C2C12 myoblasts and RAW264.7 macrophages were obtained from Shanghai Fuheng Biotechnology Co., Ltd. (Shanghai, China; Cat. Nos. FH0345 and FH0328, respectively). Both cell lines were maintained in Dulbecco’s modified Eagle’s medium (DMEM; Gibco, Thermo Fisher Scientific, Waltham, MA, USA) supplemented with 10% fetal bovine serum (Gibco) and 1% penicillin–streptomycin (C0222, Beyotime) in a humidified incubator at 37 °C with 5% CO_2_. Myogenic differentiation was induced as previously described [27]. Briefly, when C2C12 cells reached approximately 90% confluence, the growth medium was replaced with differentiation medium consisting of DMEM supplemented with 2% horse serum (S9050, Solarbio, Beijing, China) and 1% penicillin–streptomycin. The cells were differentiated for 4 days, with the differentiation medium replaced every 24 h.

RAW264.7 macrophages were assigned to the Control, HGPA, and HGPA + Ful groups. Control macrophages were maintained in control medium containing 5.5 mM glucose, whereas the other groups were exposed to HGPA (25.5 mM glucose and 200 μM palmitic acid) in the presence or absence of fullerol (20 μg/mL) for 24 h. After treatment, the culture medium was removed, and the cells were washed three times with PBS. Donor-cell numbers and viability were then determined in parallel wells subjected to the same treatments, as described in Section 2.16. Fresh drug-free complete medium was then added to the remaining identically treated cultures, and the cells were cultured for an additional 24 h. The conditioned medium was normalized to the corresponding viable donor-cell number before being applied to C2C12 myoblasts for 24 h, with an identical final medium volume maintained across all recipient cultures. Mitochondrial membrane potential, DHE fluorescence, and Fe^2+^ levels were subsequently evaluated.

### 2.4. Establishment of the Glucolipotoxicity Model and Fullerol Treatment Schedules

To establish the glucolipotoxicity model, C2C12 myoblasts were exposed to high-glucose medium (25.5 mM glucose) containing 200 μM palmitic acid for 24 h (HGPA). Palmitic acid was dissolved in DMSO to prepare a 200 mM stock solution. The stock solution was diluted 1:1000 in culture medium to obtain a final PA concentration of 200 μM and a final DMSO concentration of 0.1%. An equivalent concentration of DMSO was included in the corresponding vehicle-control groups. Cells maintained in medium containing 5.5 mM glucose served as the control. For the component-control experiments, cells were additionally treated with 20 mM mannitol in 5.5 mM glucose medium (Mannitol), 25.5 mM glucose alone (HG), or 200 μM PA in 5.5 mM glucose medium (PA).

For the time-matched treatment-schedule experiments summarized in Table 1, cells were assigned to the Control, HGPA, Con-Ful, and Post-Ful groups. All groups were maintained for a total of 48 h and analyzed at the same experimental endpoint.

For the time-matched treatment-schedule experiments summarized in Table 2, cells were assigned to the Control, HGPA, Con-Ful, Post-Ful-24 h, and Post-Ful-72 h groups. Samples used for endpoint analyses were collected at the common 96-h endpoint. Specifically, samples for qRT-PCR analysis were collected at 96 h before the induction of differentiation. Separately, MYHC protein expression was assessed in parallel cultures subjected to the same 96-h treatment schedules, followed by an additional 4 days of culture in differentiation medium.

### 2.5. Cell Viability Assay

The viability of C2C12 myoblasts and RAW264.7 macrophages exposed to the indicated concentrations of fullerol was quantified using Cell Counting Kit-8 (C0037, Beyotime) according to the manufacturer’s protocol. Cells were seeded in 96-well plates at 3000 cells/well and treated with fullerol for 24, 48, or 96 h. At each time point, the medium was replaced with 100 μL of fresh complete medium containing 10% CCK-8 reagent. After incubation for 1 h at 37 °C in the dark, absorbance at 450 nm was measured using an Infinite M200 Pro microplate reader (Tecan, Männedorf, Switzerland). For pharmacological rescue experiments, C2C12 myoblasts were exposed to HGPA for 24 h in the absence or presence of the ferroptosis inhibitors ferrostatin-1 (Fer-1, 10 μM) or liproxstatin-1 (Lip-1, 5 μM), the apoptosis inhibitor Z-VAD-FMK (Z-VAD, 10 μM), or the necroptosis inhibitor necrostatin-1 (Nec-1, 10 μM). Cell viability was subsequently determined using the CCK-8 procedure described above.

### 2.6. Live/Dead Cell Staining

The cytocompatibility of fullerol was assessed using a Live/Dead assay kit (C2015S, Beyotime) according to the manufacturer’s instructions. C2C12 and RAW264.7 cells were exposed to the indicated concentrations of fullerol for 1 and 4 days. After staining, cells were imaged using an inverted fluorescence microscope (DMi8, Leica Microsystems, Wetzlar, Germany). Viable and membrane-compromised cells exhibited green and red fluorescence, respectively. Identical acquisition settings were used for all experimental groups.

### 2.7. DHE Staining

Intracellular superoxide-associated fluorescence was evaluated using dihydroethidium (DHE; S0063, Beyotime). Following the indicated treatments, C2C12 myoblasts were incubated with DHE working solution for 30 min at 37 °C in the dark. After three washes with PBS, the nuclei were counterstained with DAPI for 5 min at room temperature. The cells were washed again with PBS, and fluorescence images were immediately acquired using a DMi8 fluorescence microscope. All images were captured using identical acquisition settings, and the mean fluorescence intensity was quantified using ImageJ software.

### 2.8. Ferrous Ion Staining

Intracellular ferrous iron (Fe^2+^) was detected using a RhoNox-6 fluorescent probe (S1070S, Beyotime). Following the indicated treatments, C2C12 myoblasts cultured in 24-well plates were washed three times with serum-free medium and incubated with RhoNox-6 working solution for 30 min at 37 °C in the dark. The nuclei were subsequently counterstained with DAPI. Fluorescence images were immediately acquired using a DMi8 fluorescence microscope. Images were obtained using identical settings across all groups, and the mean fluorescence intensity was quantified using ImageJ software (version 1.54p, National Institutes of Health, Bethesda, MD, USA).

### 2.9. Mitochondrial Membrane Potential Assay

Mitochondrial membrane potential was assessed using a JC-1 Mitochondrial Membrane Potential Assay Kit (C2003S, Beyotime) according to the manufacturer’s instructions. Following the indicated treatments, C2C12 myoblasts were washed twice with PBS and incubated with JC-1 staining working solution for 20 min at 37 °C in the dark. The cells were subsequently washed twice with PBS and immediately examined using a fluorescence microscope (DMi8, Leica Microsystems). Images were acquired using identical settings across all groups. Mitochondrial membrane potential was quantified as the ratio of red to green fluorescence intensity using ImageJ software. A lower red-to-green fluorescence ratio indicated mitochondrial membrane depolarization.

### 2.10. ABTS Radical Scavenging Test

The antioxidant capacity of fullerol was evaluated using an ABTS-based total antioxidant capacity assay kit (S0119, Beyotime). Fullerol solutions ranging from 5 to 80 μg/mL were mixed with ABTS working solution according to the manufacturer’s instructions. After incubation, absorbance at 734 nm was measured using an Infinite M200 Pro microplate reader. The ABTS radical-scavenging rate was calculated relative to the vehicle control after blank correction.

### 2.11. Hemolysis Assay

Fresh mouse erythrocytes were isolated by centrifugation and washed repeatedly with sterile PBS until the supernatant became clear. The erythrocyte suspension was incubated with the indicated concentrations of fullerol at 37 °C. PBS and deionized water served as the negative and positive controls, respectively. After centrifugation, the absorbance of the supernatant was measured using a microplate reader. The hemolysis rate was calculated as follows: hemolysis (%) = (A_sample − A_negative)/(A_positive − A_negative) × 100%.

### 2.12. RNA Extraction and Quantitative Real-Time PCR Analysis

C2C12 cells were seeded in six-well plates at 1 × 10^5^ cells/well and cultured for 24 h. Following the indicated treatments, total RNA was extracted from cells or TA muscle using TRIzol reagent (R0016, Beyotime) according to the manufacturer’s protocol. Complementary DNA was synthesized using PrimeScript RT Master Mix (RR036A, Takara, Shiga, Japan), and quantitative real-time PCR was performed using SYBR Green qPCR Mix (D7262, Beyotime Biotechnology). Relative gene expression was calculated using the 2^−ΔΔCt^ method, with Gapdh as the reference gene. Primer sequences are listed in Table 3.

### 2.13. Western Blot

Cells were lysed in RIPA buffer (P0013B, Beyotime) supplemented with 1% protease and phosphatase inhibitor cocktail (P1045, Beyotime). Following incubation at 4 °C for 20 min, samples were subjected to additional mechanical disruption to improve lysis efficiency. Lysates were mixed with 5× SDS-PAGE loading buffer (P0015, Beyotime) to a final concentration of 1× and heated at 99 °C for 5 min. Proteins were separated by SDS-PAGE and transferred onto polyvinylidene fluoride (PVDF) membranes (Millipore, Burlington, MA, USA). Membranes were blocked with 5% nonfat milk and incubated with primary antibodies overnight at 4 °C (MYHC, MYOG, GPX4, ACSL4, GAPDH, 1:1000), followed by the appropriate secondary antibodies (1:1000) for 1 h at room temperature. Protein bands were visualized using an enhanced chemiluminescence reagent (New Cell & Molecular Biotech Co., Ltd., Suzhou, China).

### 2.14. Immunofluorescence Staining

Following treatment, cells cultured in 24-well plates were washed with PBS and fixed with 4% paraformaldehyde (P0099, Beyotime) for 15 min. Cells were permeabilized with 0.2% Triton X-100 (P0096, Beyotime) for 10 min and blocked with 5% bovine serum albumin for 1 h at room temperature. Cells were incubated overnight at 4 °C with primary antibodies against ACSL4, GPX4, KI67, CCNA2, P21, γ-H2AX, CCR7, CD206, TNF-α, or SOD1 (1:100), followed by incubation with the appropriate fluorophore-conjugated secondary antibodies (1:200) for 1 h at room temperature in the dark. Nuclei were counterstained with DAPI. Images were acquired using a DMi8 fluorescence microscope under identical acquisition settings for each marker. Mean fluorescence intensity was quantified using ImageJ after background subtraction.

### 2.15. GSH and Malondialdehyde (MDA) Assay

Intracellular GSH and MDA levels were measured using GSH (BC1175) and MDA (BC0025) assay kits (Solarbio, Beijing, China), respectively. Cells were homogenized in the extraction solution provided with each kit and centrifuged at 8000× *g* at 4 °C. For GSH measurement, the absorbance of the supernatant was measured at 412 nm using an Infinite M200 Pro microplate reader. For MDA measurement, the supernatant was mixed with the reaction reagents and incubated in a boiling water bath for 1.5 h. After centrifugation at 10,000× *g* at room temperature, the absorbance of the resulting supernatant was measured at 532 and 600 nm.

### 2.16. ELISA and Conditioned-Medium Normalization

At the end of the 24-h macrophage treatment, donor-cell numbers and viability were determined in parallel wells subjected to the same treatments. Following treatment removal and PBS washing, cells from the parallel wells were harvested, mixed with trypan blue, and analyzed using a Countess II Automated Cell Counter (Thermo Fisher Scientific, Waltham, MA, USA). Viable and non-viable cells were distinguished by trypan-blue exclusion, and only viable donor-cell counts were used for conditioned-medium normalization. Fresh drug-free complete medium was then added to the remaining identically treated macrophage cultures, and conditioned medium was collected after an additional 24 h.

The volume of conditioned medium applied to recipient C2C12 myoblasts was adjusted according to the viable donor-cell number so that each recipient culture received conditioned medium corresponding to an equivalent of 1 × 10^7^ viable macrophages. Fresh drug-free complete medium was added as necessary to ensure an identical final volume in all recipient cultures.

IL-6 and TNF-α levels were measured in aliquots of the same post-wash conditioned-medium preparations used to treat C2C12 myoblasts. Cytokine concentrations were measured using ELISA kits (EK206 and EK282, respectively; Multi Sciences, Hangzhou, China) according to the manufacturer’s instructions. Briefly, standards and samples were added to antibody-precoated plates and incubated with the corresponding detection reagents. Following washing, TMB substrate was added for color development, and the reaction was terminated with stop solution. Absorbance was measured at 450 nm using an Infinite M200 Pro microplate reader, and cytokine concentrations were calculated from the standard curves, normalized to the corresponding viable donor-cell numbers, and expressed per 1 × 10^7^ viable cells.

### 2.17. C11-BODIPY 581/591

Cellular lipid peroxidation was assessed using C11-BODIPY 581/591 staining (S0043S, Beyotime). Following treatment, cells were washed with PBS and incubated with C11-BODIPY 581/591 (2 μM) at 37 °C for 30 min in the dark. Cells were then washed three times with PBS and immediately imaged using a DMi8 fluorescence microscope. Green fluorescence from the oxidized probe and red fluorescence from the reduced probe were acquired under identical imaging settings across all groups. Fluorescence intensities were quantified using ImageJ.

### 2.18. Animals and Experimental Design

#### 2.18.1. Animals and Housing Conditions

All animal procedures were approved by the Laboratory Animal Ethics Committee of Shanghai Ninth People’s Hospital, Shanghai Jiao Tong University School of Medicine (Approval No. SH9H-2026-A1292-1) and were conducted in accordance with institutional guidelines. Eight-week-old male C57BL/6J mice weighing 20–24 g were purchased from Shanghai Jihui Laboratory Animal Care Co., Ltd. (Shanghai, China). The mice were specific-pathogen-free, immunocompetent, genetically unmodified, and had not undergone any previous experimental procedures. Before group allocation, the mice were acclimatized for 5 days in the specific-pathogen-free animal facility of Shanghai Ninth People’s Hospital. Mice were housed at 22 ± 3 °C under a 12-h light/dark cycle with free access to food and water.

#### 2.18.2. Group Allocation, Sample Size, and Blinding

At study initiation, mice were randomly assigned to four groups (n = 6 per group): control, Ful, diabetic (DM), and fullerol-treated diabetic (DM + Ful). Group allocation was performed by drawing lots. All animals were maintained under standardized housing conditions, and treatments and measurements were performed according to the same predefined procedures. No additional measures were used to randomize cage location or the order of treatments and measurements. Investigators responsible for group allocation and treatment administration were aware of group assignments, whereas histological imaging and quantitative outcome assessment were performed by an investigator blinded to group allocation. Group assignments were revealed before statistical analysis.

The group size of six animals per group was determined based on previous studies using comparable diabetic mouse models and skeletal muscle models [5,28], while minimizing animal use in accordance with the 3Rs principle. No formal a priori sample-size calculation was performed. No additional prespecified inclusion or exclusion criteria were applied after group allocation. No animals or data points were excluded from the analyses, and all 24 mice were included in the final analyses (n = 6 per group). The exact sample size for each analysis is reported in the corresponding figure legend.

#### 2.18.3. Induction of the Diabetic Model

To establish a type 2 diabetes mellitus model, mice in the DM and DM + Ful groups were fed a high-fat diet and received consecutive streptozotocin injections according to the induction protocol [5]. Mice in the control and Ful groups were fed a standard diet containing 10% kcal from fat (D12450B, Research Diets, New Brunswick, NJ, USA), whereas mice in the DM and DM + Ful groups were fed a high-fat diet containing 60% kcal from fat (D12492, Research Diets) throughout the 12-week experimental period. After 4 weeks of dietary intervention, mice in the DM and DM + Ful groups were fasted overnight and intraperitoneally injected with streptozotocin (STZ; 25 mg/kg) once daily for 4 consecutive days. STZ was freshly dissolved in ice-cold 0.1 M citrate buffer, and mice in the control and Ful groups received an equivalent volume of citrate buffer.

#### 2.18.4. Fullerol Administration and Sample Collection

Fullerol administration was initiated on the first day of dietary intervention and continued throughout the 12-week experimental period. Mice in the Ful and DM + Ful groups received bilateral intramuscular injections of fullerol into the tibialis anterior (TA) muscles at a volume of 20 μL per muscle (20 μg/mL) once every 3 days. Control and untreated diabetic mice received equal-volume bilateral injections of sterile saline following the same schedule. All injections were performed aseptically by trained investigators using the minimum required volume to reduce discomfort.

Animals were monitored daily for general condition, activity, food and water intake, and injection-site abnormalities. Humane endpoints included severe lethargy, inability to access food or water, or markedly impaired mobility; no animals reached these endpoints or experienced procedure-related adverse events.

Local intramuscular administration was selected to deliver fullerol directly to the target skeletal muscle while limiting systemic exposure. At the experimental endpoint, mice were deeply anesthetized by inhalation of 2% isoflurane and euthanized by cervical dislocation while under anesthesia. The TA muscles were rapidly excised, weighed, and either fixed for histological analysis or stored at −80 °C for molecular analysis. Relative TA muscle weight was calculated by normalizing TA muscle weight to body weight. The animal-study outcome measures included body weight and fasting blood glucose as indicators of the diabetic phenotype; relative TA muscle weight and myofiber cross-sectional area as indicators of muscle atrophy; and the expression of ferroptosis-, myogenesis-, and muscle atrophy-associated genes in TA muscle. No single primary outcome measure was prespecified for sample-size determination because no formal a priori sample-size calculation was performed.

### 2.19. Histological Analysis

TA muscles were fixed in 4% paraformaldehyde for 48 h, dehydrated, embedded in paraffin, and transversely sectioned at a thickness of 5 μm. Sections were stained with hematoxylin and eosin and imaged by bright-field microscopy. Myofiber cross-sectional area was quantified from transverse sections obtained from a standardized region of the TA muscle using ImageJ. Multiple non-overlapping fields were analyzed for each mouse, and the mean value for each mouse was treated as one biological replicate. For each mouse, all clearly delineated myofibers within 3 non-overlapping fields were analyzed.

### 2.20. Tissue Immunofluorescence Staining Analysis

Immunofluorescence staining was performed to assess 4-hydroxynonenal (4-HNE) and F4/80 in tissue sections. Paraffin-embedded sections were deparaffinized, rehydrated, and subjected to antigen retrieval. After blocking nonspecific binding, sections were incubated with primary antibodies against 4-HNE or F4/80 (1:50) overnight at 4 °C, followed by the corresponding fluorophore-conjugated secondary antibodies (1:100) for 1 h at room temperature in the dark. Sections were washed with PBS and mounted with antifade mounting medium. Images were acquired under identical settings across all groups. For each mouse, 3 non-overlapping fields from comparable TA muscle regions were analyzed. Field-level measurements were averaged to obtain one value per mouse, and the mouse was used as the experimental unit. Mean fluorescence intensity was quantified using ImageJ after background subtraction. F4/80-positive macrophage abundance was quantified as the number of F4/80-positive cells per unit tissue area.

### 2.21. Metabolic Assessment and Hindlimb Grip Strength Measurement

At week 12, mice were fasted for 12 h before metabolic measurements. Fasting blood glucose was measured in tail-vein blood using an Accu-Chek Performa glucometer (Roche Diagnostics). Blood samples were also collected, and serum insulin concentrations were measured using a mouse insulin ELISA kit (SEKM-0387, Solarbio, Beijing, China) according to the manufacturer’s instructions. Hindlimb grip strength was assessed using a grip strength meter (Ugo Basile, Gemonio, Italy). Mice were gently held by the loose skin behind the neck and positioned on a grid inclined at approximately 30°, allowing both hind paws to grip the grid while preventing forepaw contact. Each mouse was pulled steadily along the plane of the grid until its hind paws released, and the peak force was recorded. Five trials were performed per mouse, with a 30 s rest interval between trials, and the maximum force was used for analysis.

### 2.22. Statistical Analysis

All experiments were performed with three independent biological replicates unless otherwise specified. Data are presented as the mean ± standard deviation (SD). For cell experiments, independent experiments performed using separately cultured and treated cells were considered biological replicates. Multiple wells, microscopic fields, or individual cells obtained from the same cell culture were considered technical observations rather than independent biological replicates. For quantitative image-based analyses, 3 non-overlapping fields were analyzed per independent experiment. Measurements from these fields or cells were averaged to generate one value for each independent experiment, which was used as the experimental unit for statistical analysis. For animal experiments, each mouse was considered one biological replicate.

Because formal normality testing has limited reliability with only three biological replicates, normality testing was not used to determine the statistical analysis of the in vitro datasets. For in vitro comparisons between two groups, an unpaired two-tailed Student’s *t*-test was used. For comparisons among three or more groups, one-way analysis of variance followed by Tukey’s multiple-comparisons test was used. These analyses were performed using the independent experiment-level means, and the results were interpreted cautiously given the limited number of biological replicates. Individual biological replicate values are displayed in the graphs.

For animal datasets, normality and homogeneity of variance were assessed using the Shapiro–Wilk and Brown–Forsythe tests, respectively. For comparisons among three or more groups, normally distributed data with homogeneous variance were analyzed by one-way analysis of variance followed by Tukey’s multiple-comparisons test; otherwise, the Kruskal–Wallis test followed by Dunn’s multiple-comparisons test was applied.

Statistical analyses were performed using GraphPad Prism 10.0 (GraphPad Software, Boston, MA, USA). All tests were two-sided, and *p* < 0.05 was considered statistically significant. Exact *p* values are presented for statistically significant comparisons. Non-significant comparisons are not annotated except where the absence of a significant difference is directly relevant to result interpretation or validation of control experiments.

## 3. Results

### 3.1. Glucolipotoxicity Induces Ferroptosis-Associated Injury and Alters Proliferation- and Myogenesis-Associated Markers

**Figure 1:**
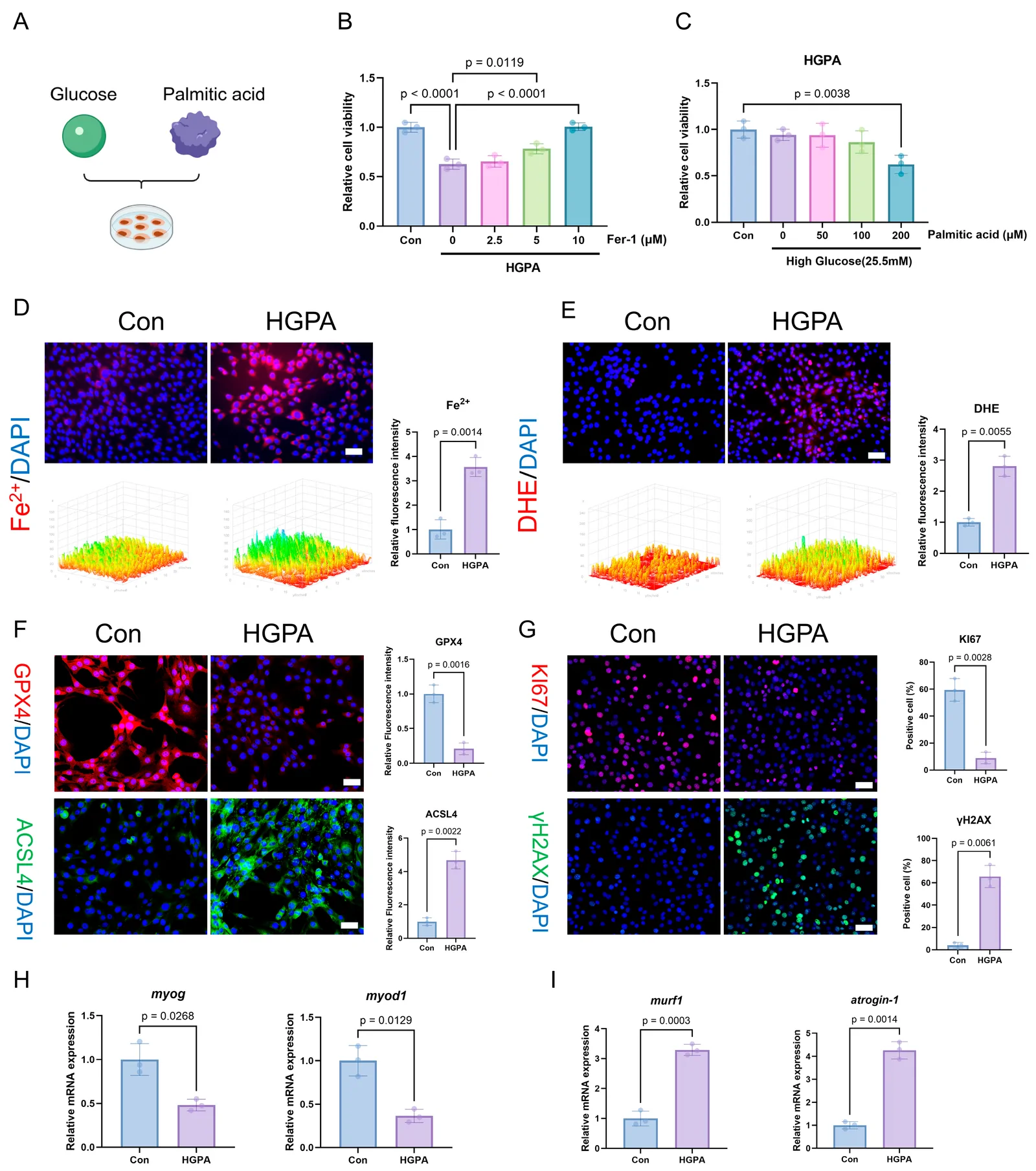
Glucolipotoxicity induces ferroptosis-associated injury and alters proliferation- and myogenesis-associated markers. (**A**) Schematic illustration of high-glucose and palmitic acid (HGPA) induction in C2C12 myoblasts. (Created with BioGDP.com). (**B**) CCK-8 analysis of C2C12 myoblast viability following exposure to increasing concentrations of palmitic acid (PA) under high-glucose conditions (25.5 mM) for the indicated durations. Medium containing 5.5 mM glucose served as the normal control. (**C**) CCK-8 analysis of myoblast viability following co-treatment with vehicle or the indicated concentrations of ferrostatin-1 (Fer-1). (**D**) Representative fluorescence images and quantitative analysis of intracellular Fe^2+^ accumulation following 24 h of HGPA exposure. Scale bar: 50 μm. (**E**) Representative DHE staining and quantitative analysis of intracellular superoxide anion after 24 h of HGPA exposure. Scale bar: 50 μm. (**F**) Representative immunofluorescence images and quantitative analysis of ACSL4 and GPX4 expression under control and HGPA conditions. Scale bar: 50 μm. (**G**) Representative immunofluorescence images and quantitative analysis of the proliferation-associated protein KI67 and the DNA damage marker γ-H2AX following 24 h of HGPA exposure. Scale bar: 50 μm. (**H**,**I**) qRT-PCR analysis of the myogenic regulatory genes *myod1* and *myog* and the muscle atrophy-associated genes *murf1* and *atrogin-1* following 24 h of HGPA exposure. Data are presented as the mean ± SD from three independent biological experiments (n = 3). For image-based analyses, three non-overlapping fields were analyzed per experiment and averaged to generate one value for each biological replicate. Exact *p* values are shown in the corresponding figures.

To model diabetic glucolipotoxicity, C2C12 myoblasts were exposed to high glucose and palmitic acid (HGPA) (Figure 1A). Cell viability decreased in a PA concentration-dependent manner. After 24 h of exposure to 200 μM PA, myoblast viability decreased to 62.25% of the control level. Higher PA concentrations resulted in insufficient numbers of viable myoblasts for subsequent experiments. Therefore, treatment with 200 μM PA was selected for the following experiments (Figure 1B). The ferroptosis inhibitor ferrostatin-1 (Fer-1) dose-dependently rescued the HGPA-induced reduction in cell viability (Figure 1C), consistent with the involvement of ferroptosis-associated injury in HGPA-induced cytotoxicity. To further pharmacologically characterize HGPA-induced cell injury, rescue experiments were performed using the ferroptosis inhibitors ferrostatin-1 (Fer-1) and liproxstatin-1 (Lip-1), the apoptosis inhibitor Z-VAD-FMK, and the necroptosis inhibitor necrostatin-1 (Nec-1). Fer-1 and Lip-1 significantly restored cell viability, whereas no significant protective effects were observed with Z-VAD-FMK or Nec-1 under the tested conditions (Figure S1A). Because the activity of Z-VAD-FMK and Nec-1 was not independently confirmed using pathway-specific positive controls, these negative findings were not used to exclude the potential involvement of apoptosis or necroptosis.

Consistent with this finding, HGPA exposure markedly increased intracellular Fe^2+^ accumulation and DHE fluorescence, indicating disrupted iron homeostasis and enhanced oxidative stress (Figure 1D,E). Immunofluorescence staining revealed increased ACSL4 and decreased GPX4 expression in HGPA-treated myoblasts (Figure 1F). We subsequently assessed lipid peroxidation using C11-BODIPY staining. The results showed that HGPA exposure significantly increased intracellular lipid ROS levels (Figure S1B). To distinguish the individual effects of high glucose (HG) and palmitic acid (PA) from the potential influence of glucose-induced hyperosmolarity, we included an osmotically matched mannitol control and evaluated HG and PA separately. Neither mannitol nor HG alone significantly reduced cell viability, whereas PA caused a moderate reduction. In contrast, combined HGPA exposure produced a more pronounced decrease in cell viability. Neither mannitol nor HG alone significantly altered MDA or GSH levels, whereas PA moderately increased MDA and decreased GSH. Combined HGPA exposure produced more pronounced changes in both indicators (Figure S1C,D). Collectively, these results indicate that the detrimental effects of HGPA were not attributable to altered osmolarity and were more pronounced than those induced by either HG or PA alone.

We next examined whether HGPA affected cellular markers associated with myoblast proliferation and myogenesis. Immunofluorescence staining showed decreased KI67 together with increased γH2AX accumulation in HGPA-treated cells, indicating reduced proliferative activity and an enhanced DNA-damage response (Figure 1G). In parallel, qPCR analysis showed that HGPA decreased the expression of the myogenic regulatory genes *myod1* and *myog* while increasing the expression of the muscle atrophy-associated genes *murf1* and *atrogin-1* (Figure 1H). After an identical 4-day period in differentiation medium, MYHC protein expression was unaffected by mannitol or HG alone, moderately reduced by PA, and markedly suppressed by combined HGPA exposure (Figure S1E). These results indicate that glucolipotoxicity not only induces ferroptosis-associated injury but also adversely alters proliferation- and myogenesis-associated molecular markers in C2C12 myoblasts.

### 3.2. Characterization, Cytocompatibility, and Antioxidant Activity of Fullerol

**Figure 2:**
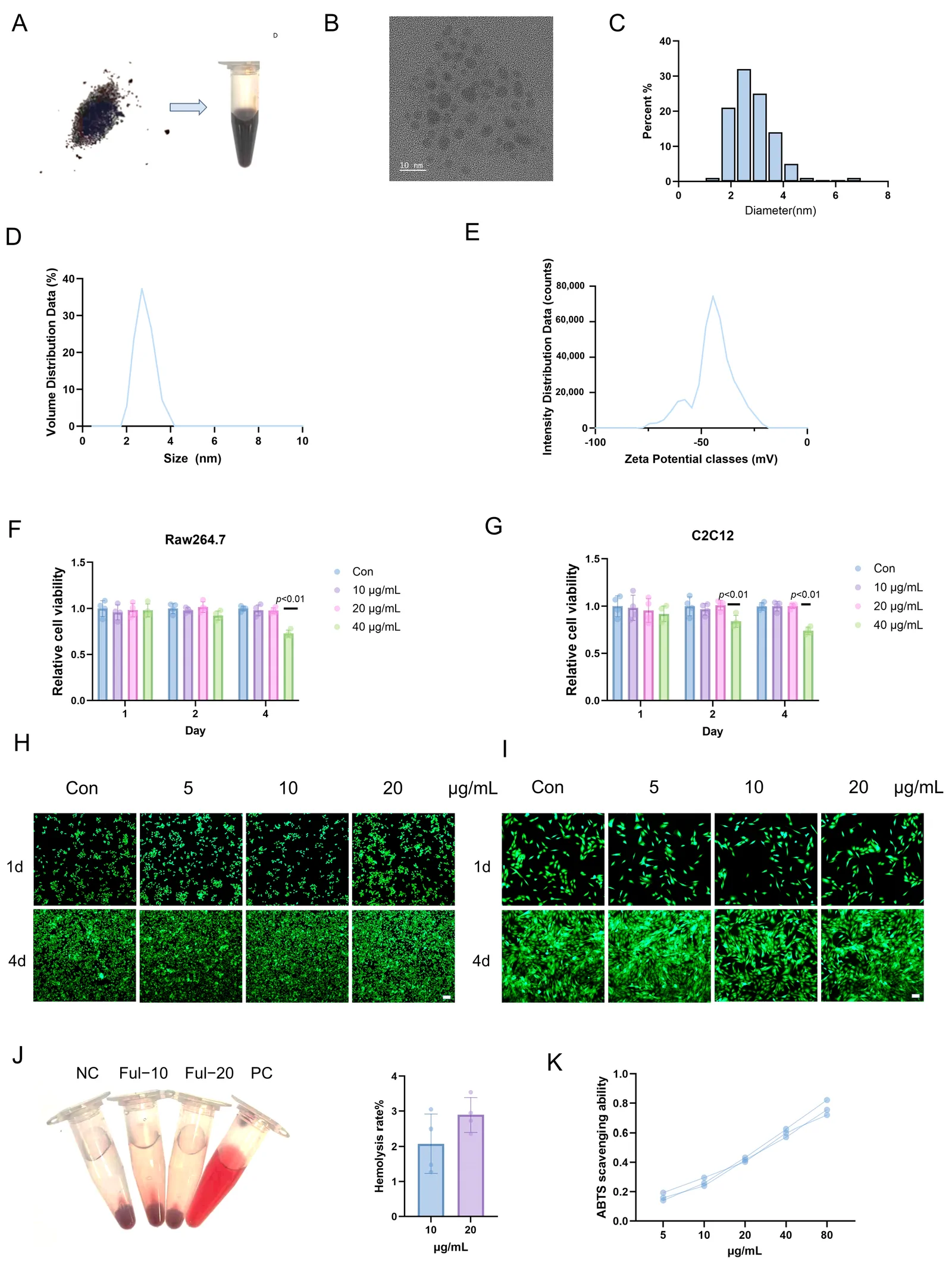
Characterization, cytocompatibility, and antioxidant activity of fullerol. (**A**) Representative photographs of fullerol as a solid powder and after reconstitution as an aqueous dispersion (30 mg/mL). (**B**,**C**) Representative transmission electron microscopy (TEM) image of fullerol and the corresponding particle size distribution. (**D**) Dynamic light scattering (DLS) analysis of the hydrodynamic size distribution of fullerol in aqueous dispersion. (**E**) Zeta potential distribution of fullerol. (**F**) CCK-8 analysis of RAW264.7 macrophage viability following exposure to the indicated concentrations of fullerol for 1, 2, or 4 days. (**G**) CCK-8 analysis of C2C12 myoblast viability following exposure to the indicated concentrations of fullerol for 1, 2, or 4 days. (**H**) Representative Live/Dead staining images of RAW264.7 macrophages following treatment with the indicated concentrations of fullerol. (**I**) Representative Live/Dead staining images of C2C12 myoblasts following treatment with the indicated concentrations of fullerol. (**J**) Representative photographs and quantitative analysis of red blood cell hemolysis following exposure to the indicated concentrations of fullerol. (**K**) ABTS-based total antioxidant capacity assay showing the concentration-dependent radical-scavenging activity of fullerol. Data are presented as the mean ± SD from three independent biological experiments (n = 3). For image-based analyses, three non-overlapping fields were analyzed per experiment and averaged to generate one value for each biological replicate. Exact *p* values are shown in the corresponding figures.

Fullerol was first characterized to determine its properties. The material appeared as a fine black powder and formed a dark aqueous dispersion after reconstitution (Figure 2A). Transmission electron microscopy revealed approximately spherical particles with a diameter of approximately 2.83 nm (Figure 2B,C), while dynamic light scattering showed a hydrodynamic diameter of approximately 2.74 nm at 20 μg/mL (Figure 2D). The dispersion exhibited a zeta potential of approximately −44.5 mV, indicating a strongly negative surface charge (Figure 2E). In parallel, DLS and zeta-potential measurements were performed at the experimental concentration of 20 μg/mL in culture medium, revealing a mean hydrodynamic diameter of 2.88 nm and a mean zeta potential of −43.8 mV (Figure S2A,B). These results indicate that fullerol retained its small hydrodynamic size and strongly negative surface charge under the relevant culture conditions, consistent with previous reports [29].

The cytocompatibility of fullerol was subsequently evaluated in RAW264.7 macrophages and C2C12 myoblasts. CCK-8 assays showed that fullerol did not significantly reduce cell viability at the tested concentrations over 1, 2, or 4 days (Figure 2F,G). Live/Dead staining similarly revealed high cell viability with minimal cell death in both cell types (Figure 2H,I). In addition, hemolysis remained below 5% across all tested concentrations, indicating favorable hemocompatibility (Figure 2J). The cell-free antioxidant activity of fullerol was evaluated using an ABTS-based total antioxidant capacity assay. Fullerol exhibited concentration-dependent radical-scavenging activity, increasing from 16.4% at the lowest tested concentration to 76.5% at 80 μg/mL (Figure 2K).

Finally, to assess whether fullerol directly interfered with the absorbance- or fluorescence-based assays, we performed additional interference controls. Fullerol was added either to the CCK-8 reagent alone or to the cell-generated CCK-8 reaction product. The addition of fullerol did not significantly alter absorbance at 450 nm under either condition (Figure S2C). Cells stained with DHE, RhoNox-6, or JC-1 were subsequently exposed to vehicle or fullerol for 2 min immediately before fluorescence imaging (Figure S2D,E). No significant changes were observed in DHE, RhoNox-6 or JC-1 fluorescence intensity. These results indicate that, despite its intrinsic radical-scavenging activity, fullerol did not produce detectable immediate interference with the CCK-8, DHE, RhoNox-6, or JC-1 assay readouts at the concentration and exposure duration tested.

Collectively, these findings demonstrate that fullerol possesses concentration-dependent antioxidant activity together with favorable in vitro cytocompatibility and hemocompatibility.

### 3.3. Fullerol Attenuates Ferroptotic Injury Under Concurrent and Post-HGPA Treatment Schedules

**Figure 3:**
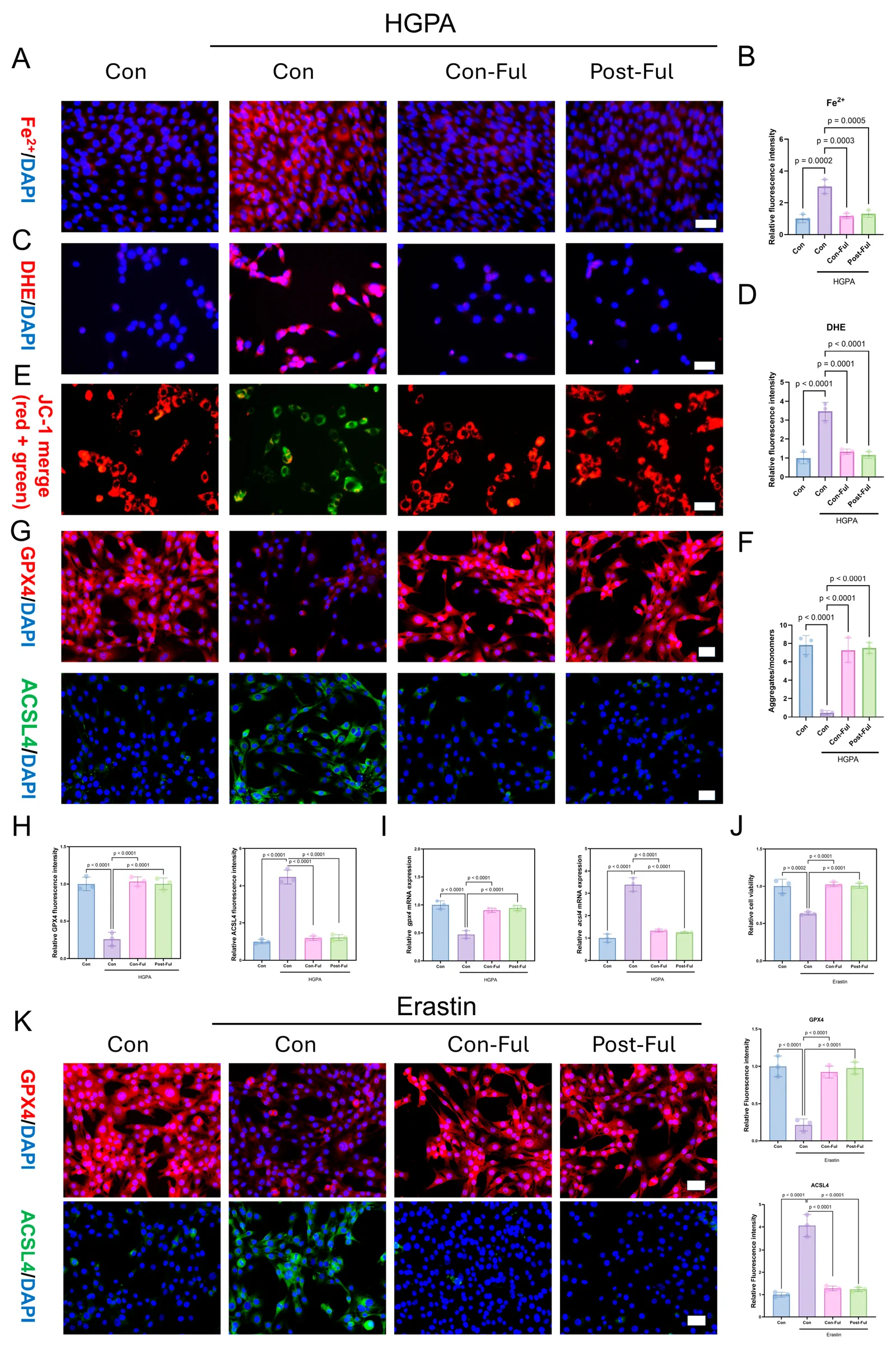
Fullerol attenuates ferroptotic injury under concurrent and post-HGPA treatment schedules. In the Con-Ful group, C2C12 myoblasts were simultaneously treated with HGPA and fullerol for 24 h. In the Post-Ful group, cells were exposed to HGPA for 24 h and subsequently treated with fullerol for an additional 24 h. (**A**,**B**) Representative fluorescence images and quantitative analysis of intracellular Fe^2+^ accumulation. (**C**,**D**) Representative DHE staining and quantitative analysis of superoxide-associated oxidative stress levels. (**E**,**F**) Representative JC-1 staining and quantitative analysis of mitochondrial membrane potential (ΔΨm). (**G**,**H**) Representative immunofluorescence images and quantitative analysis of ACSL4 and GPX4 protein expression. (**I**) Relative mRNA expression of *acsl4* and *gpx4* determined by quantitative real-time PCR. (**J**) CCK-8 analysis of C2C12 myoblast viability following erastin (10 μM) exposure under the indicated fullerol treatment schedules. (**K**) Representative immunofluorescence images and quantitative analysis of ACSL4 and GPX4 protein expression following treatment with erastin alone or erastin combined with fullerol. Scale bars: 50 μm. Data are presented as the mean ± SD from three independent biological experiments (n = 3). For image-based analyses, three non-overlapping fields were analyzed per experiment and averaged to generate one value for each biological replicate, and exact *p* values are shown in the corresponding panels.

We next investigated whether fullerol attenuated HGPA-induced ferroptotic injury under different treatment schedules. In the concurrent-treatment group (Con-Ful), fullerol was administered together with HGPA, whereas in the post-treatment group (Post-Ful), fullerol was administered for 24 h after the initial HGPA challenge. The complete treatment timelines are provided in Table 1. Both treatment schedules significantly reduced HGPA-induced intracellular Fe^2+^ accumulation (Figure 3A,B). DHE staining similarly showed that concurrent and post-HGPA fullerol treatment reduced superoxide-associated oxidative stress accumulation (Figure 3C,D). Moreover, JC-1 staining demonstrated that both treatment schedules mitigated the HGPA-induced loss of mitochondrial membrane potential (Figure 3E,F and Figure S3A). We subsequently measured intracellular MDA and GSH levels. Both Con-Ful and Post-Ful significantly reduced MDA levels and increased GSH levels (Figure S3B). Immunofluorescence staining showed that HGPA increased ACSL4 and decreased GPX4 protein expression, whereas both fullerol treatment schedules attenuated these alterations (Figure 3G,H). Consistent with the protein-level findings, quantitative real-time PCR analysis showed that concurrent and post-HGPA fullerol treatment suppressed *acsl4* expression and restored *gpx4* expression (Figure 3I). Collectively, these findings indicate that fullerol attenuates HGPA-induced iron accumulation, oxidative stress, mitochondrial dysfunction, and ferroptosis-associated molecular alterations. The protective effect of fullerol against ferroptosis-associated injury was further evaluated using erastin as a pharmacological ferroptosis inducer. The CCK-8 assay showed that both fullerol treatment schedules significantly rescued the erastin-induced reduction in cell viability (Figure 3J). Immunofluorescence staining further showed that erastin increased ACSL4 and decreased GPX4 protein expression, whereas fullerol co-treatment attenuated these alterations (Figure 3K).

Together, these results support the protective effect of fullerol against ferroptosis-associated injury induced by either glucolipotoxic stress or a classical ferroptosis inducer. Notably, fullerol remained effective in attenuating ferroptosis-associated alterations even when administered after prior HGPA exposure.

### 3.4. Concurrent Fullerol Treatment More Effectively Preserves Myogenesis-Associated Responses in Myoblasts

Because both concurrent and post-treatment fullerol administration attenuated ferroptotic injury, we next examined whether prolonged fullerol treatment after HGPA exposure could restore proliferation- and myogenesis-associated molecular markers. In the Con-Ful group, fullerol was administered during HGPA exposure. In the Post-Ful groups, cells were exposed to HGPA for 24 h and subsequently treated with fullerol for either 24 h or 72 h. All groups were analyzed at a common 96-h endpoint. The HGPA group was exposed to HGPA for 24 h and subsequently maintained in fullerol-free control medium for 72 h, thereby serving as the recovery-only control. The complete treatment timelines are provided in Table 2.

**Figure 4:**
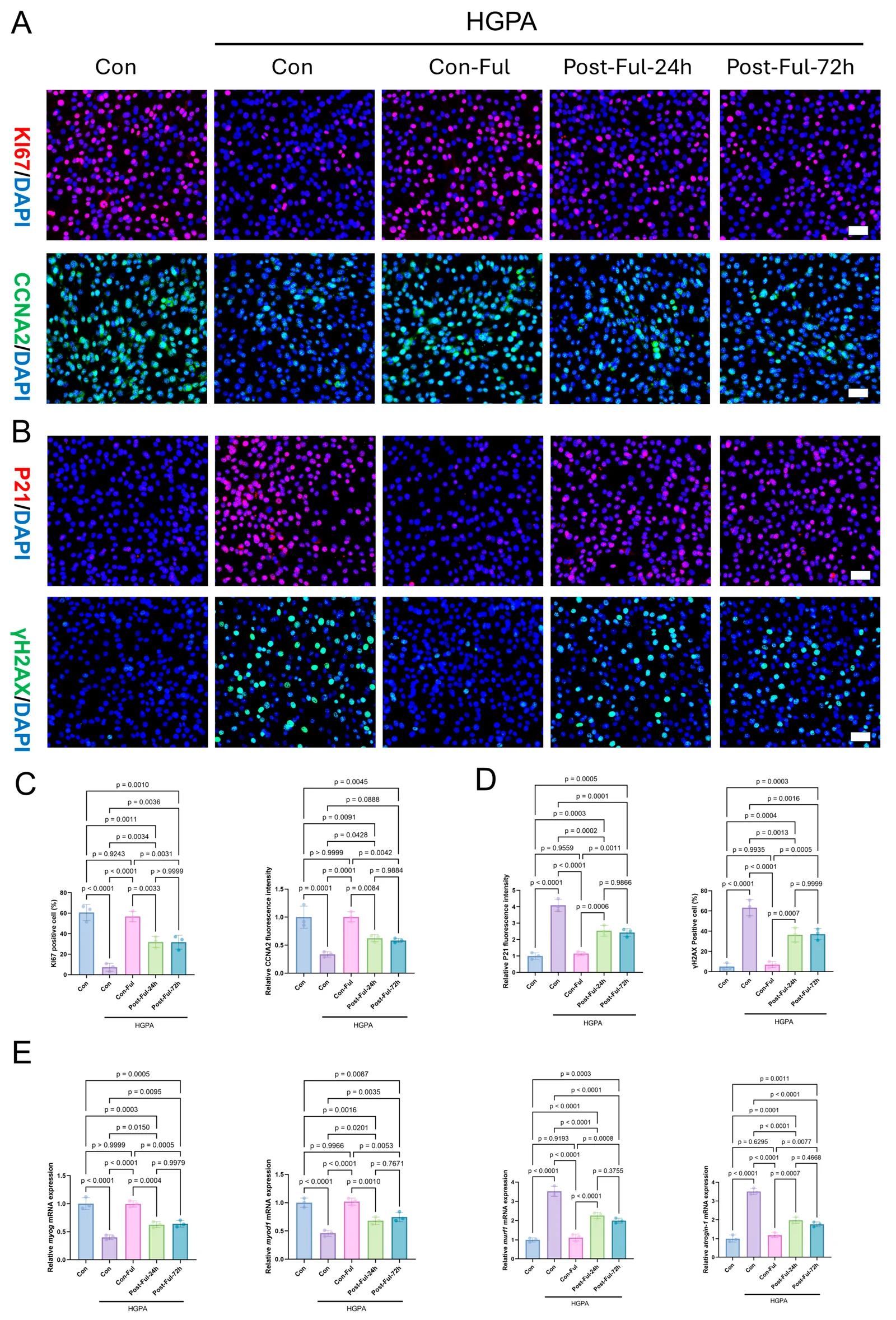
Concurrent fullerol treatment more effectively preserves myogenesis-associated responses in myoblasts. In the Con-Ful group, C2C12 myoblasts were treated simultaneously with HGPA and fullerol for 24 h. In the post-treatment groups, cells were first exposed to HGPA for 24 h and subsequently treated with fullerol for either 24 h (Post-Ful-24 h) or 72 h (Post-Ful-72 h). All samples shown in panels A–E were collected at the common 96-h endpoint before the induction of differentiation. (**A**,**C**) Representative immunofluorescence images and quantitative analysis of the proliferation-associated proteins KI67 and CCNA2 following the indicated treatments. Scale bar: 50 μm. (**B**,**D**) Representative immunofluorescence images and quantitative analysis of P21 and γ-H2AX expression. Scale bar: 50 μm. (**E**) qRT-PCR analysis of the myogenic regulatory genes *myod1* and *myog* and the muscle atrophy-associated genes *murf1* and *atrogin-1*. Data are presented as the mean ± SD from three independent biological experiments (n = 3). For image-based analyses, three non-overlapping fields were analyzed per experiment and averaged to generate one value for each biological replicate. Exact *p* values are shown in the corresponding figures.

Immunofluorescence staining showed that HGPA markedly reduced the expression of the proliferation-associated proteins KI67 and CCNA2. Concurrent fullerol treatment largely preserved the expression of both proteins. By contrast, the Post-Ful-24 h group showed only partial recovery, and extending fullerol treatment to 72 h did not restore KI67 or CCNA2 expression to the levels observed with concurrent treatment (Figure 4A,C). These findings indicate that the limited recovery following post-treatment was not simply attributable to an insufficient duration of fullerol exposure.

HGPA also increased P21 and γH2AX expression. These alterations were markedly attenuated by concurrent fullerol treatment but were only partially reduced in the Post-Ful-24 h group. Notably, P21 and γH2AX remained elevated even after 72 h of post-treatment fullerol exposure (Figure 4B,D). Given that P21 and γH2AX are associated with cell-cycle inhibition and DNA damage, respectively, their persistent elevation indicates that fullerol treatment initiated after HGPA-induced injury had been established was insufficient to fully reverse these stress-associated cellular alterations, even when the treatment duration was extended.

We further evaluated genes associated with myogenic differentiation and muscle atrophy. qPCR analysis showed that concurrent fullerol treatment largely restored the HGPA-suppressed expression of *myod1* and *myog*, while inhibiting the HGPA-induced upregulation of *murf1* and *atrogin-1*. Fullerol treatment initiated after HGPA exposure only partially restored *myod1* and *myog* expression and only partially suppressed *murf1* and *atrogin-1* expression. Extending the post-treatment period to 72 h did not achieve effects comparable to those observed with concurrent treatment (Figure 4E). After the identical differentiation period, MYHC protein expression was assessed by Western blot. Concurrent fullerol treatment largely restored MYHC protein expression, whereas fullerol treatment initiated after HGPA exposure resulted in only partial recovery (Figure S4).

Collectively, these findings demonstrate that attenuation of ferroptosis-associated injury does not necessarily coincide with complete recovery of proliferation- and myogenesis-associated molecular markers. Once glucolipotoxic injury has been established, prolonged fullerol treatment can attenuate ferroptosis-associated changes but cannot fully restore proliferation- and myogenesis-associated marker expression within the observed period.

### 3.5. Fullerol Modulates Macrophage Phenotypic Markers and Redox–Inflammatory Responses

**Figure 5:**
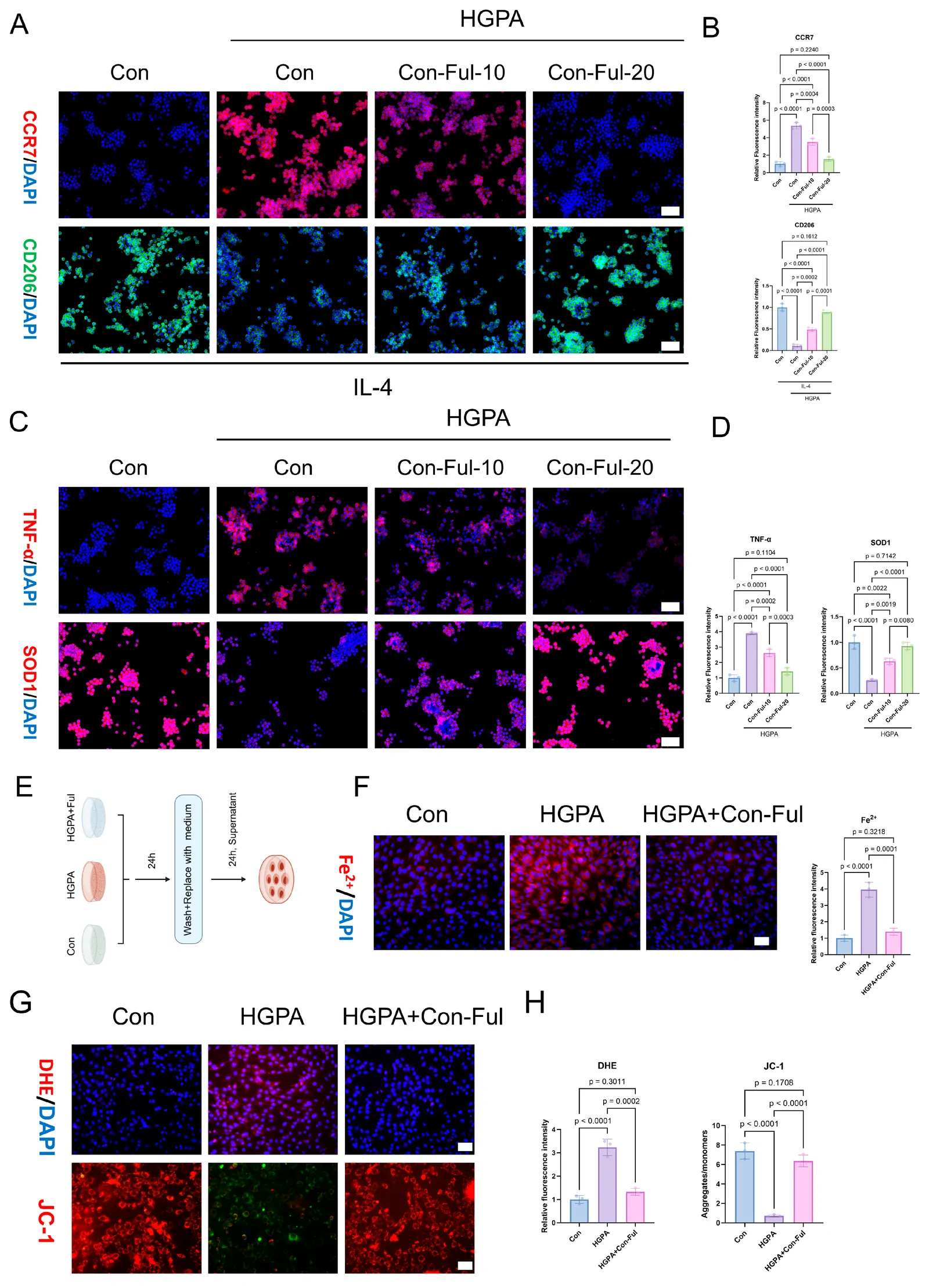
Fullerol modulates macrophage phenotypic markers and redox–inflammatory responses. (**A**,**B**) Representative immunofluorescence images (**A**) and quantitative analyses (**B**) of CCR7, a marker associated with pro-inflammatory macrophage responses, and CD206, a marker associated with anti-inflammatory/tissue-remodeling phenotypes. Scale bar: 50 μm. Ful-10/20:10/20 μg/mL fullerol. (**C**,**D**) Representative immunofluorescence images (**C**) and quantitative analyses (**D**) of the pro-inflammatory cytokine TNF-α and the antioxidant enzyme SOD1. Scale bar: 50 μm. (**E**) Schematic illustration of the macrophage-conditioned-medium experiment. Following treatment, RAW264.7 macrophages were washed, and donor-cell numbers and viability were determined in parallel wells using trypan-blue exclusion and a Countess II Automated Cell Counter. The remaining identically treated macrophages were then cultured in fresh drug-free complete medium for 24 h. The resulting conditioned medium was collected, normalized to an equivalent of 1 × 10^7^ viable donor macrophages, and applied to C2C12 myoblasts for 24 h, with an identical final medium volume maintained across all groups. Created with BioGDP.com. (**F**) Representative fluorescence images and quantitative analysis of intracellular Fe^2+^ accumulation in C2C12 myoblasts. (**G**,**H**) Representative DHE and JC-1 staining and quantitative analysis. Scale bars: 50 μm. Data are presented as the mean ± SD from three independent biological experiments (n = 3). For image-based analyses, three non-overlapping fields were analyzed per experiment and averaged to generate one value for each biological replicate, and exact *p* values are shown in the corresponding panels.

Macrophages are important regulators of skeletal muscle homeostasis and atrophy [30]. To determine whether fullerol modulated macrophage responses to glucolipotoxic stress, RAW264.7 macrophages were exposed to HGPA and evaluated by immunofluorescence staining. HGPA increased CCR7, a marker associated with pro-inflammatory macrophage responses, and decreased CD206, a marker associated with anti-inflammatory and tissue-remodeling phenotypes. Fullerol treatment attenuated these alterations, as evidenced by decreased CCR7 and increased CD206 immunoreactivity (Figure 5A,B). To further characterize the inflammatory response, we measured the expression of inflammation-related genes in macrophages. HGPA significantly upregulated *tnf-α*, *il6*, and *il-1β* expression while downregulating *il10* expression, whereas fullerol treatment significantly attenuated these changes (Figure S5A). Consistently, ELISA analysis of aliquots from the same post-wash conditioned-medium preparations subsequently used to treat C2C12 myoblasts showed that fullerol attenuated the HGPA-induced increases in TNF-α and IL-6 levels (Figure S5B). These findings indicate that fullerol shifts macrophage phenotypic marker expression toward a less pro-inflammatory profile under glucolipotoxic conditions.

Consistent with these phenotypic changes, HGPA increased TNF-α and decreased SOD1 expression in macrophages, whereas fullerol attenuated both alterations (Figure 5C,D). To evaluate macrophage-derived paracrine effects, the treatments were removed, and the macrophages were washed and subsequently cultured in fresh drug-free medium. The conditioned medium was normalized to the viable donor-macrophage number before being applied to C2C12 myoblasts. Each recipient culture received conditioned medium corresponding to an equivalent of 1 × 10^7^ viable donor macrophages, with an identical final medium volume maintained across all groups (Figure 5E). Conditioned medium from HGPA-stimulated macrophages induced Fe^2+^ accumulation (Figure 5F) and increased DHE fluorescence and mitochondrial membrane potential loss in C2C12 myoblasts (Figure 5G,H and Figure S5C). In contrast, conditioned medium from macrophages treated with HGPA plus fullerol markedly attenuated these effects.

Collectively, these findings indicate that fullerol modulates macrophage phenotypic and redox–inflammatory responses and that conditioned medium from HGPA-plus-fullerol-treated macrophages induced weaker oxidative, iron-related, and mitochondrial alterations in myoblasts. Although these findings support an altered macrophage-derived paracrine environment, they do not identify the specific soluble factors responsible for these effects.

### 3.6. Localized Fullerol Administration During Model Development Attenuates Diabetic Muscle Atrophy In Vivo

Finally, we evaluated the protective effects of localized fullerol administration during model development. At the end of the 12-week modeling period, diabetic mice exhibited significantly increased body weight, fasting blood glucose and insulin levels compared with non-diabetic controls, consistent with the establishment of a type 2 diabetes-like metabolic phenotype (Figure S6A) [31]. Local intramuscular fullerol administration was initiated on day 1 and continued during the subsequent development of diabetic muscle atrophy.

**Figure 6:**
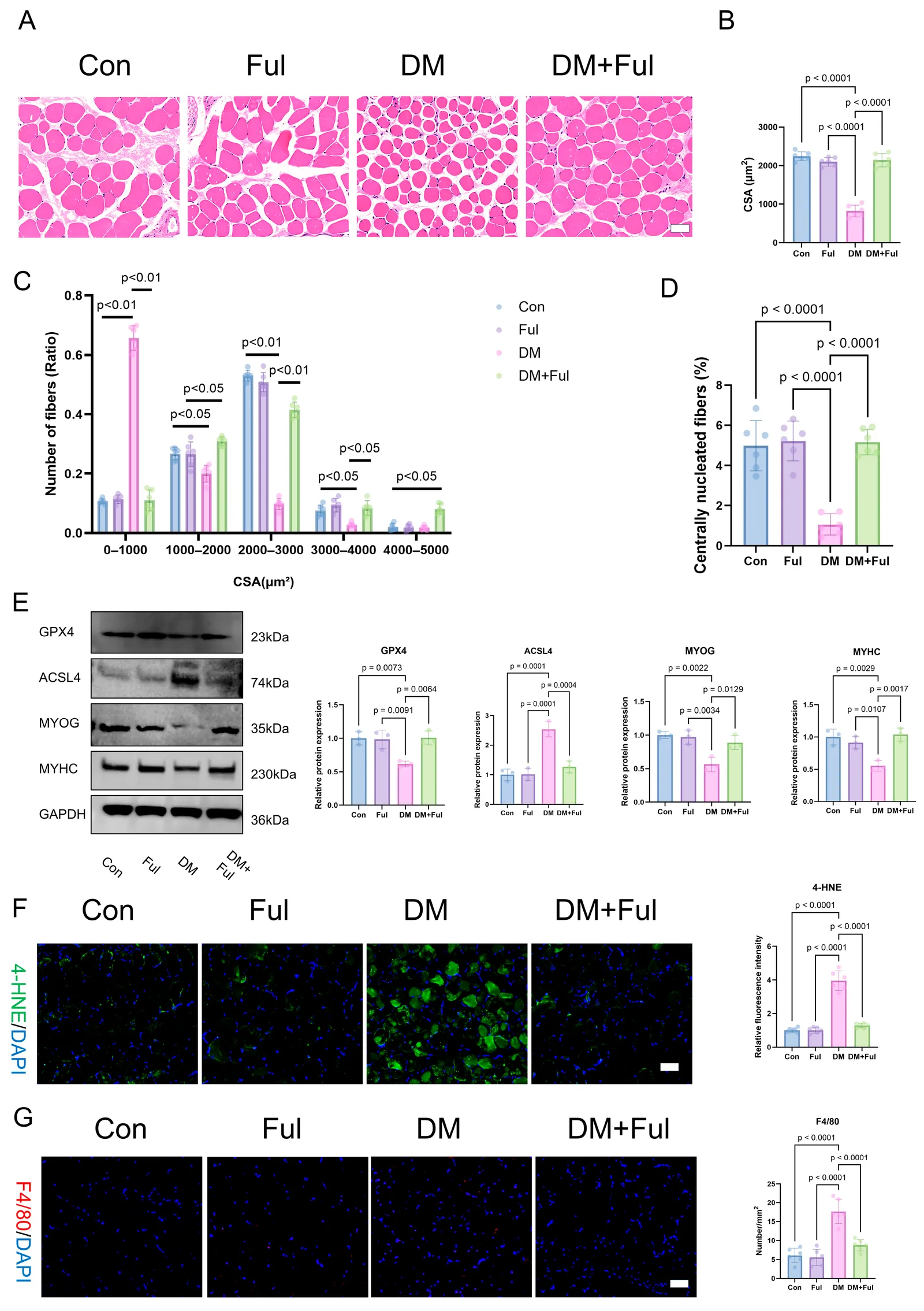
Localized fullerol administration during model development attenuates diabetic muscle atrophy in vivo. (**A**) Representative hematoxylin and eosin (H&E)-stained cross-sections of tibialis anterior (TA) muscle from the indicated groups. (**B**) Quantification of mean myofiber cross-sectional area (CSA). (**C**) Frequency distribution of individual myofiber cross-sectional areas (CSA; μm^2^). (**D**) Quantification of the percentage of centrally nucleated myofibers. (**E**) Representative Western blot images and analysis of MYHC, MYOG, GPX4, and ACSL4 protein expression in TA muscle, with GAPDH used as the loading control. (**F**) Representative immunofluorescence images and quantitative analysis of 4-HNE in TA muscle. (**G**) Representative F4/80 immunofluorescence images and quantification of F4/80-positive macrophage density in TA muscle. Scale bars: 50 μm. Data are presented as the mean ± SD (n = 6 mice per group). For image-based analyses, three non-overlapping fields were analyzed per mouse and averaged to generate one value for each mouse. Con, non-diabetic vehicle-treated mice; Ful, non-diabetic mice receiving fullerol alone; DM, untreated diabetic mice; DM + Ful, diabetic mice receiving localized fullerol administration during model development.

Absolute and relative TA muscle weights showed corresponding patterns, with significantly greater values in fullerol-treated diabetic mice than in untreated diabetic mice (Figure S6B). Further assessment of hindlimb grip strength showed a partial recovery following fullerol treatment (Figure S6C). Histological analysis revealed a marked reduction in tibialis anterior (TA) myofiber cross-sectional area in diabetic mice. Localized fullerol administration during model development significantly attenuated this reduction and preserved myofiber size (Figure 6A,B). Likewise, analysis of the myofiber cross-sectional-area distribution showed a shift toward larger myofiber cross-sectional areas following fullerol treatment. The proportion of centrally nucleated myofibers was also restored toward control levels (Figure 6C,D). qRT-PCR analysis further showed that diabetic muscle exhibited reduced *myod1* and *myog* expression accompanied by increased *murf1* expression. Localized fullerol administration during model development reversed these changes (Figure S6D). Diabetic muscle exhibited reduced MYHC and MYOG protein expression, whereas fullerol treatment attenuated these reductions (Figure 6E). These changes were consistent with the protective effects of concurrent fullerol treatment observed in HGPA-treated C2C12 myoblasts. Consistently, Western blot analysis showed that fullerol administration increased GPX4 expression and decreased ACSL4 expression in diabetic muscle (Figure 6E). Immunofluorescence staining for 4-HNE showed that fullerol treatment significantly reduced lipid peroxidation in skeletal muscle (Figure 6F). We also evaluated macrophage abundance in skeletal muscle. F4/80 immunofluorescence staining showed an increased abundance of F4/80-positive macrophages in diabetic muscle, which was significantly reduced by fullerol treatment (Figure 6G). Consistently, qPCR analysis showed that fullerol attenuated the diabetes-associated upregulation of the inflammatory genes *tnf-α*, *il6*, and *il-1β* in whole-muscle tissue, supporting a reduction in the local inflammatory environment (Figure S6E). Notably, fullerol administration alone did not significantly alter the measured parameters, suggesting no detectable adverse effects on these outcomes at the tested dose and treatment schedule. These findings indicate that localized fullerol treatment reduced ferroptosis-associated alterations in diabetic skeletal muscle.

Taken together, localized fullerol administration during model development preserved myofiber size and muscle weight, improved the expression of myogenesis-associated genes and proteins, suppressed atrophy-associated gene expression, and attenuated ferroptosis-associated alterations in diabetic skeletal muscle. These findings support the in vivo protective efficacy of fullerol when administered throughout diabetic model development.

## 4. Discussion

Diabetic muscle atrophy has traditionally been attributed primarily to impaired insulin signaling and an imbalance between protein synthesis and degradation [6,32]. Insulin is also a major regulator of nutrient and electrolyte homeostasis in skeletal muscle. It promotes GLUT4 translocation and glucose uptake, while insulin signaling and amino acid availability cooperatively regulate muscle protein synthesis and proteolysis [33,34]. Insulin also stimulates cellular potassium uptake, primarily through activation of the Na^+^/K^+^-ATPase [35]. Accordingly, impaired muscle insulin action may reduce glucose utilization, disturb amino acid and protein metabolism, and alter potassium homeostasis, thereby contributing to the loss of skeletal muscle mass. In the present HFD/STZ model, the fasting glucose and serum insulin findings indicate disturbed systemic glucose–insulin homeostasis. Nevertheless, intracellular glucose, amino acid, and potassium transport, GLUT4 translocation, and muscle insulin-signaling activity were not directly examined. Therefore, the extent to which alterations in these pathways contributed to the observed muscle phenotype remains to be determined. The present findings support a broader view in which the regenerative compartment of skeletal muscle is also vulnerable to the diabetic metabolic environment. Previous studies have shown that diabetes impairs muscle progenitor cells and that macrophage-derived factors under diabetic conditions may disrupt muscle homeostasis [36,37]. Diet-induced obesity likewise delays muscle regeneration and promotes defective tissue remodeling [38]. More specifically, elevated extracellular adenosine and AMP in diabetic mice has been shown to impair muscle satellite-cell activation through the ENT–ADK–AMPK–mTORC1 axis, whereas diet-induced obesity disrupts satellite-cell activation and muscle repair through altered hepatocyte growth factor signaling [39,40]. Our study adds a redox-related dimension to this framework by showing that HGPA exposure induced iron accumulation, lipid peroxidation, GSH depletion, mitochondrial depolarization, and GPX4/ACSL4 alterations concurrently with reduced cell viability and adverse changes in proliferation- and myogenesis-associated markers. Accordingly, diabetic muscle loss may involve not only the atrophy and dysfunction of existing myofibers but also disturbances in the cellular programs required for muscle maintenance and repair. However, because the present in vitro experiments used an immortalized C2C12 myoblast line, these findings do not directly demonstrate depletion or dysfunction of the satellite-cell pool in vivo. Moreover, although MYHC Western blot provided protein-level evidence of altered myogenesis-associated responses, myotube formation, fusion index, multinucleated-myotube formation, and myotube diameter were not assessed. The in vitro findings should therefore not be interpreted as direct evidence of functional muscle regeneration [41].

An emerging body of evidence supports a close relationship between iron metabolism, ferroptosis, and muscle regeneration. C2C12 myotubes are susceptible to erastin-induced ferroptosis mediated by disruption of cystine-glutamate antiporter system [42,43]. More importantly, satellite cell-specific deletion of transferrin receptor 1 leads to labile iron accumulation and ferroptosis together with impaired satellite-cell proliferation, differentiation, and skeletal muscle regeneration [44]. More recent work showed that ferroptosis activation inhibited C2C12 differentiation and myotube formation, whereas modulation of the OTUB1–P62 pathway attenuated ferroptotic injury and supported myogenesis [45]. Another recent study identified an LCN2–ACOD1-dependent ferroptosis pathway that impaired C2C12 proliferation and myotube formation and delayed post-injury skeletal muscle regeneration [46]. These findings are consistent with our observation that glucolipotoxic stress induced iron accumulation, oxidative stress, mitochondrial dysfunction, and ferroptosis-associated molecular changes while suppressing proliferation- and myogenesis-associated marker profiles [47,48,49,50].

In the present study, increased C11-BODIPY oxidation and MDA levels, GSH depletion, Fe^2+^ accumulation, GPX4/ACSL4 alterations, and the rescue of cell viability by Fer-1 and Lip-1 collectively support the involvement of ferroptosis-associated injury in the cellular response to HGPA. Z-VAD-FMK and Nec-1 did not significantly restore cell viability under the tested conditions. However, because the activity of these inhibitors was not independently confirmed using pathway-specific positive controls, their lack of rescue cannot be used to determine the relative contributions of different cell-death pathways or to exclude the potential involvement of apoptosis or necroptosis. Therefore, the present pharmacological and biochemical findings support the involvement of ferroptosis-associated injury but do not establish ferroptosis as the exclusive or primary mode of cell death. Genetic validation and mitochondrial ultrastructural analysis were not performed.

This protection-versus-recovery distinction is supported by evidence that hyperglycemia and saturated fatty acids disrupt myoblast function through processes extending beyond overt cell death. In the component-control experiments, an osmotically matched mannitol control and HG alone did not significantly affect cell viability, MDA, GSH, or MYHC expression, whereas PA produced moderate alterations and combined HGPA exposure caused more pronounced effects. These results indicate that the HGPA-induced phenotype was not attributable to glucose-induced hyperosmolarity and exceeded the effect of either HG or PA alone. High glucose suppresses *myod* and *myog* expression and inhibits C2C12 differentiation through impaired AKT signaling [51]. Palmitic acid similarly reduces myoblast proliferation and myogenic-factor expression and impairs myotube formation, even at concentrations that do not induce detectable cytotoxicity [52]. These findings indicate that metabolic stress can directly alter the cellular machinery governing myogenesis independently of its lethal effects. In our model, concurrent fullerol treatment may preserve this machinery by limiting the initial oxidative and ferroptotic burden. Once HGPA-induced alterations have become established, however, suppression of ongoing ferroptotic stress may prevent additional injury without fully resetting the proliferation- and differentiation-associated cellular state.

Persistent DNA damage and cell-cycle responses provide a plausible explanation for this incomplete recovery. Activation of the DNA-damage response can antagonize MYOD-dependent myogenic program, whereas inhibition of DNA-damage signaling restores the myogenic program in human senescent fibroblasts [53]. Similarly, bleomycin-induced DNA damage increases γ-H2AX and P21, causes sustained cell-cycle arrest, and severely impairs C2C12 myotube formation despite having limited effects on cell viability [54]. In our study, the parallel reduction in KI67 and CCNA2, elevation of P21 and γ-H2AX, and suppression of *myod1* and *myog* indicate that HGPA disrupts both myoblast expansion and differentiation. Although P21 alone is not evidence of senescence because it also participates in physiological cell-cycle exit [55], its elevation together with γ-H2AX and reduced proliferative and myogenic markers supports a persistent stress-associated state. Such persistent alterations may limit the recovery of proliferation- and myogenesis-associated marker profiles even after ferroptosis-associated stress has been attenuated. Accordingly, these in vitro findings suggest that concurrent intervention is more effective than post-HGPA treatment in preserving these cellular responses.

Previous studies of fullerol further support the importance of intervention timing. Fullerol reduced oxidative stress, restored mitochondrial function, alleviated DNA damage, and suppressed cellular senescence in lens epithelial cells [56]. In a rat model of doxorubicin-induced acute hepatotoxicity, fullerenol nanoparticles administered before doxorubicin significantly improved survival, reduced ROS, and attenuated structural liver damage [57]. Consistent with this preventive paradigm, fullerol pretreatment enhanced the osteogenic potential of human adipose-derived stem cells [58]. Although these studies involved different progenitor-cell populations, they predominantly introduced fullerol before or during oxidative stress, supporting its ability to preserve cellular competence when antioxidant protection is established early. The present study extends these findings by directly comparing concurrent and post-HGPA fullerol administration in C2C12 cells. Extending post-HGPA fullerol exposure to 72 h did not reproduce the preservation of proliferation- and myogenesis-associated markers achieved by concurrent treatment. Thus, attenuation of ferroptosis-associated biochemical injury and recovery of myogenesis-associated marker profiles represent distinct biological outcomes in vitro. Whether intervention timing similarly influences the efficacy of fullerol in vivo remains to be determined.

The macrophage findings suggest an additional macrophage-associated microenvironmental contribution to myoblast injury. Macrophage responses during skeletal muscle homeostasis and repair are heterogeneous and cannot be fully represented by a binary M1/M2 classification [59,60,61,62]. A recent single-cell and muscle-organoid study identified multiple macrophage subpopulations during skeletal muscle regeneration and demonstrated that a specific HGF-producing subset promoted muscle stem-cell proliferation through HGF/MET signaling [63]. Conversely, senescent macrophages have recently been shown to promote skeletal muscle ferroptosis and muscle atrophy through paracrine disruption of redox and metabolic homeostasis [64].

In our experiments, HGPA increased CCR7 and TNF-α and decreased CD206 and SOD1 expression in macrophages. HGPA also increased *tnf-α*, *il6*, and *il-1β* expression and reduced *il10* expression, whereas fullerol attenuated these changes. Consistently, ELISA showed that fullerol reduced the HGPA-induced secretion of TNF-α and IL-6. After treatment removal and washing, cell-number-normalized conditioned medium from HGPA-stimulated macrophages increased DHE fluorescence, Fe^2+^ accumulation, and mitochondrial membrane potential loss in C2C12 myoblasts. Conditioned medium from HGPA-plus-fullerol-treated macrophages attenuated these alterations. These findings indicate that fullerol modifies macrophage-derived conditioned medium in a manner associated with reduced oxidative, iron-related, and mitochondrial stress in myoblasts. However, the responsible soluble mediators and their causal relevance in vivo remain to be determined.

The additional tissue-level analyses strengthened the connection between the cellular and animal experiments. Recent experimental work has also identified ferroptosis-related molecular alterations in diabetic skeletal muscle atrophy, supporting the potential relevance of ferroptotic injury to diabetic muscle pathology [15]. In the present study, diabetic skeletal muscle exhibited increased 4-HNE accumulation, reduced GPX4 expression, and increased ACSL4 expression, whereas fullerol attenuated these alterations. These results provide in vivo protein- and tissue-level evidence of reduced lipid peroxidation and ferroptosis-associated molecular changes following fullerol treatment. Diabetic muscle also exhibited reduced MYHC and MYOG protein expression, decreased myofiber size and muscle weight, and impaired hindlimb grip strength. The partial recovery of these outcomes following fullerol administration supports a protective effect on muscle morphology, myogenesis-associated protein expression, and general muscle function during diabetic model development.

In addition, diabetic muscle showed increased F4/80-positive macrophage abundance and elevated *tnf-α*, *il6*, and *il-1β* expression, all of which were attenuated by fullerol. These findings are consistent with a reduction in the local inflammatory environment. However, F4/80 staining does not define macrophage phenotype, and whole-muscle qPCR cannot identify whether the transcriptional changes originated from macrophages, myofibers, satellite cells, or other resident cell populations. Therefore, the in vivo findings support an association among fullerol treatment, reduced ferroptosis-associated alterations, and an attenuated inflammatory environment but do not establish a macrophage-specific causal pathway.

### Limitations

Local fullerol administration during diabetic model development preserved TA muscle weight and myofiber size and partially restored hindlimb grip strength. However, because no delayed-treatment animal group was included, the influence of intervention timing is supported only by the in vitro comparison. Generalizability is also limited by the use of only male C57BL/6J mice and the HFD/STZ model. Although group allocation and outcome assessment were randomized and blinded, respectively, no formal a priori sample-size calculation was performed, and cage location and procedural order were not randomized. In addition, grip strength alone does not comprehensively assess muscle function, additional behavioral tests were not performed and body-composition data were unavailable. Finally, intramuscular administration served as a proof-of-concept approach but may be impractical for generalized muscle atrophy. Future studies should evaluate broader functional outcomes, systemic or muscle-targeted delivery, pharmacokinetics, biodistribution, and repeated-dose safety.

## 5. Conclusions

In conclusion, at the 48-h endpoint, both concurrent and 24-h post-HGPA fullerol treatment attenuated glucolipotoxicity-induced ferroptosis-associated injury. At the separate 96-h endpoint, concurrent fullerol treatment more effectively preserved proliferation- and myogenesis-associated marker profiles, whereas post-HGPA fullerol treatment produced only partial recovery, even when continued for 72 h. MYHC protein expression assessed in parallel cultures after an additional 4 days of differentiation showed a similar pattern, with greater recovery following concurrent than post-HGPA treatment. Fullerol also modulated macrophage phenotypic markers and redox–inflammatory responses and reduced macrophage-conditioned-medium-induced oxidative stress and Fe^2+^ accumulation in myoblasts. In vivo, local fullerol administration during diabetic model development preserved myofiber size and relative muscle weight, improved myogenesis-associated gene and protein expression, reduced 4-HNE accumulation and F4/80-positive macrophage abundance, and attenuated atrophy- and ferroptosis-associated molecular alterations. Collectively, these findings support the protective potential of local fullerol administration during the development of diabetic muscle atrophy. The timing-dependent comparison was restricted to the in vitro C2C12 model, and whether delayed fullerol administration can reverse established diabetic muscle atrophy remains to be determined.

## Figures and Tables

**Table 1:** Experimental groups and time-matched treatment schedules used for the experiments. HGPA: 25.5 mM glucose and 200 μM palmitic acid; Ful: 20 μg/mL fullerol. All groups were time-matched and analyzed at the same experimental endpoint.

Group	0–24 h	24–48 h	Endpoint
Control	Control medium	Control medium	Analysis at 48 h
HGPA	HGPA	Fullerol-free control medium	Analysis at 48 h
HGPA+ Con-Ful	HGPA + fullerol	Fullerol-free control medium	Analysis at 48 h
HGPA + Post-Ful	HGPA	Fullerol	Analysis at 48 h

**Table 2:** Experimental groups and treatment schedules. HGPA: 25.5 mM glucose and 200 μM palmitic acid; Ful: 20 μg/mL fullerol. All groups were time-matched and analyzed at the same experimental endpoint.

Group	0–24 h	24–48 h	48–96 h	Endpoint
Control	Control medium	Control medium	Control medium	Analysis at 96 h
HGPA	HGPA	Fullerol-free control medium	Fullerol-free control medium	Analysis at 96 h
HGPA + Con-Ful	HGPA + fullerol	Fullerol-free control medium	Fullerol-free control medium	Analysis at 96 h
HGPA + Post-Ful-24 h	HGPA	Fullerol	Fullerol-free control medium	Analysis at 96 h
HGPA + Post-Ful-72 h	HGPA	Fullerol	Fullerol	Analysis at 96 h

**Table 3:** Primer sequences used for quantitative real-time PCR.

Gene	Forward Primer (5′–3′)	Reverse Primer (5′–3′)
*Acsl4*	CTTCCTCTTAAGGCCGGGAC	TCTCTTTGCCATAGCGTTTTTAGAT
*Gpx4*	GGAGCCCATTCCTGAACCTTT	ATGCACACGAAACCCCTGT
*Myod1*	TGCTCTGATGGCATGATGGATT	CTATGCTGGACAGGCAGTCG
*Myog*	GTCCCAACCCAGGAGATCATT	AGTTGGGCATGGTTTCGTCT
*Murf1*	ACCTGCTGGTGGAAAACATC	CTTCGTGTTCCTTGCACATC
*Atrogin-1*	AGAGAGGCAGATTCGCAAGCGT	TGCAAAGCTGCAGGGTGACCC
*Tnf-α*	CCCTCACACTCACAAACCAC	ACAAGGTACAACCCATCGGC
*Il6*	CTGCAAGAGACTTCCATCCAG	AGTGGTATAGACAGGTCTGTTGG
*Il10*	AGCCTTATCGGAAATGATCCAGT	GGCCTTGTAGACACCTTGGT
*Il-1β*	TGCCACCTTTTGACAGTGATG	TGATGTGCTGCTGCGAGATT
*Gapdh*	AGGTCGGTGTGAACGGATTTG	GGGGTCGTTGATGGCAACA

## Data Availability

The original contributions presented in this study are included in the article. Further inquiries can be directed to the corresponding authors.

## Supplementary Materials

The following supporting information can be downloaded at https://www.mdpi.com/article/10.3390/life16101746/s1.
