# Supplementary material for: Fullerol Attenuates Diabetic Muscle Atrophy and Ferroptosis-Associated Injury: Intervention Timing Influences Myogenesis-Associated Responses In Vitro
Source: Life (Basel). 2026 Oct 17;16(10):1746. doi: 10.3390/life16101746 (PMC13706367; doi:10.3390/life16101746)
Supplement: Supplementary file 1 [file life-16-01746-s001.zip › life-4556521-supplementary.pdf]

Fullerol Attenuates Diabetic Muscle Atrophy and Ferroptosis-Associated Injury: Intervention Timing Influences Myogenesis-Associated Responses In Vitro

Zengguang Wang [1,2,†], Yuxin Zhang [3,†], Chen Xu [1,2,*] and Dongyun Gu [1,2,*]

[1] Department of Orthopaedic Surgery, Shanghai Ninth People's Hospital, Shanghai Jiao Tong University School of Medicine, Shanghai 200011, China

[2] School of Biomedical Engineering and Med-X Research Institute, Shanghai Jiao Tong University, Shanghai 200011, China

[3] Department of Oral Surgery, Shanghai Ninth People's Hospital, Shanghai Jiao Tong University School of Medicine, Shanghai 200011, China

* Correspondence: 116126@sh9hospital.org.cn (C.X.); dmctgait@126.com (D.G.)

† These authors contributed equally to this work.

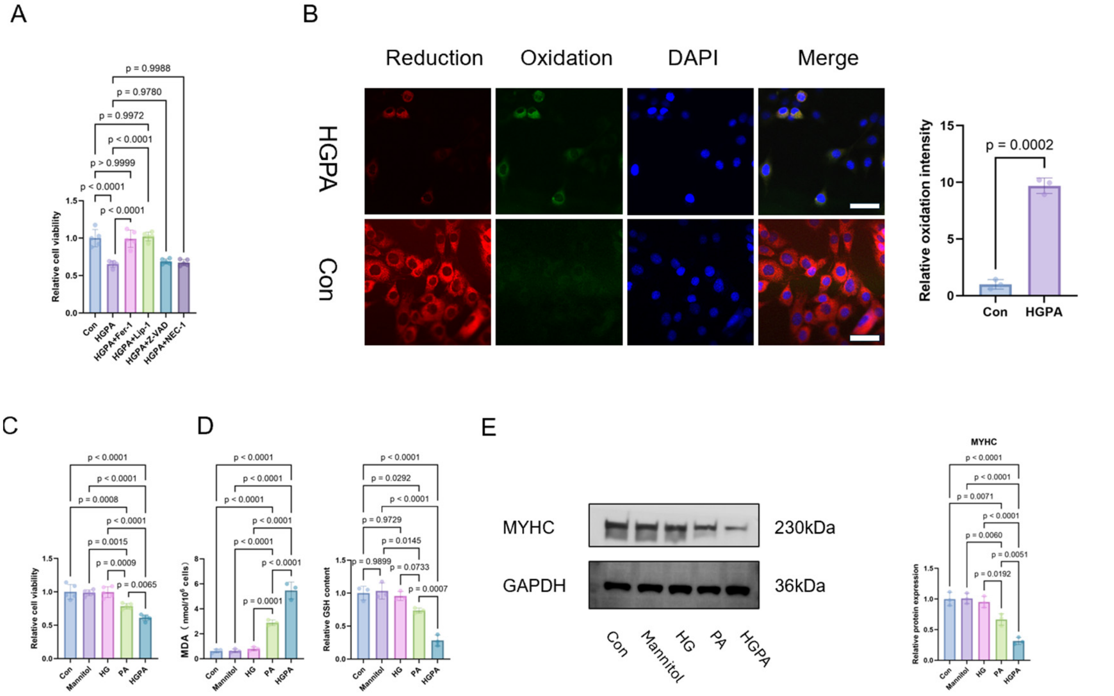

Figure S1: (A) Cell viability after HGPA exposure in the presence of the ferroptosis inhibitors ferrostatin-1 (Fer-1, 10μM) and liproxstatin-1 (Lip-1, 5μM), the apoptosis inhibitor Z-VAD-FMK (Z-VAD, 10μM), or the necroptosis inhibitor necrostatin-1 (Nec-1, 10μM). (B) Representative C11-BODIPY fluorescence images and quantitative analysis of intracellular lipid ROS levels in control and HGPA-treated myoblasts. Scale bar: 50 μm. (C) Cell viability of C2C12 myoblasts treated with mannitol, high glucose (HG), palmitic acid (PA), or combined HG and PA (HGPA). (D) Intracellular MDA and GSH levels following the indicated treatments. (E) Representative western blot images and quantitative analysis of MYHC protein expression, with GAPDH used as the loading control. Data are presented as the mean ± SD from three independent biological experiments (n = 3). For image-based analyses, three non-overlapping fields were analyzed per experiment and averaged to generate one value for each biological replicate. Exact *p* values are shown in the corresponding figures.

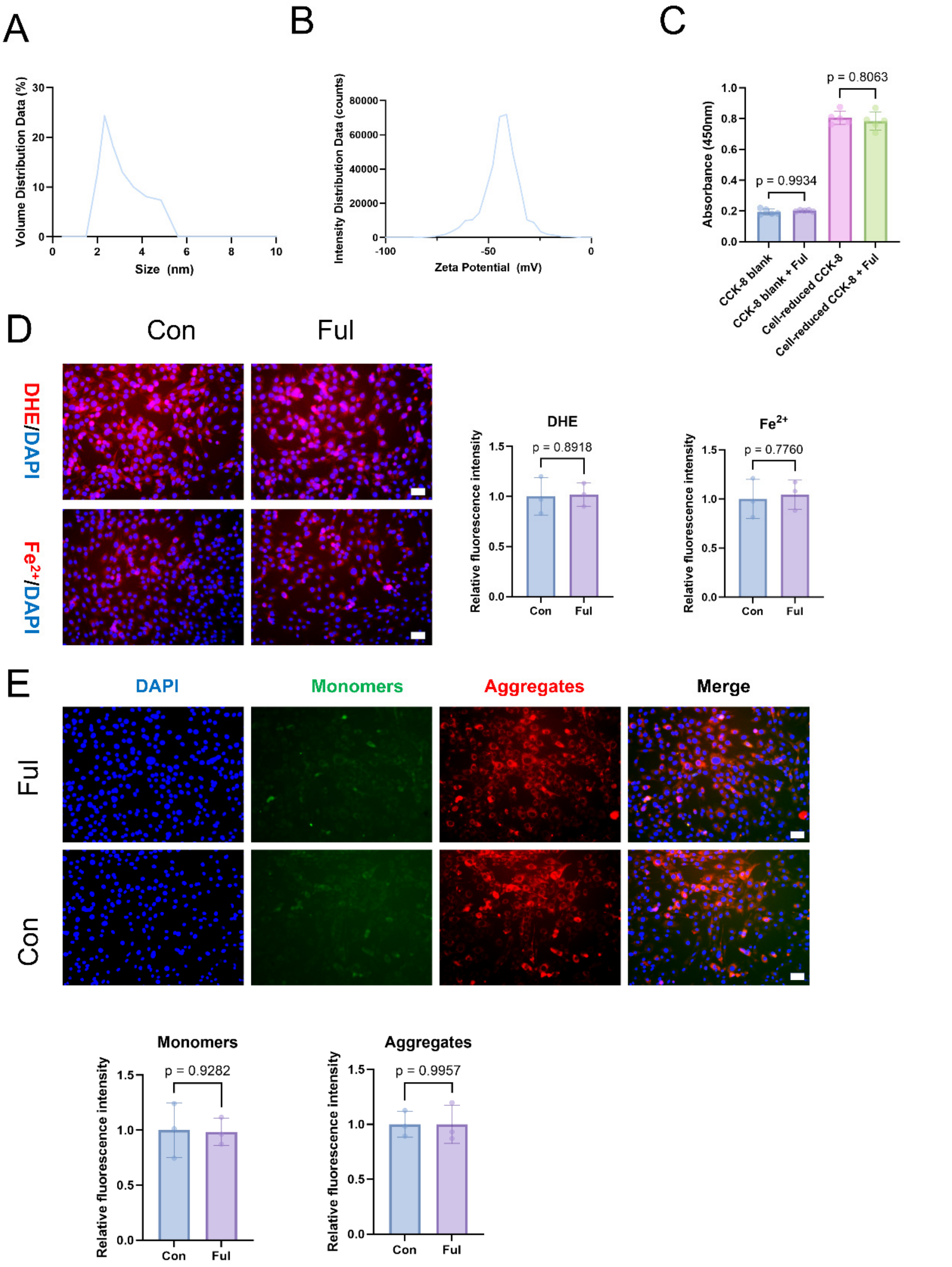

Figure S2: (A) Hydrodynamic size distribution of fullerol (20 μg/mL) in culture medium measured by dynamic light scattering (DLS), showing a mean diameter of 2.88 nm. (B) Zeta-potential distribution of fullerol in culture medium, showing a mean value of −43.8 mV. (C) Absorbance at 450 nm of CCK-8 reagent alone, CCK-8 reagent containing fullerol, the cell-generated CCK-8 reaction product, and the cell-generated reaction

product supplemented with fullerol. (D) Quantification of DHE and RhoNox-6 fluorescence following brief coincubation with or without fullerol under the corresponding assay conditions. (E) Representative JC-1 fluorescence images and quantitative analysis of the red and green fluorescence intensity following brief coincubation with or without fullerol. Scale bar: 50 μm. Data are presented as the mean ± SD from three independent biological experiments (n = 3). For image-based analyses, three non-overlapping fields were analyzed per experiment and averaged to generate one value for each biological replicate. Exact *p* values are shown in the corresponding figures.

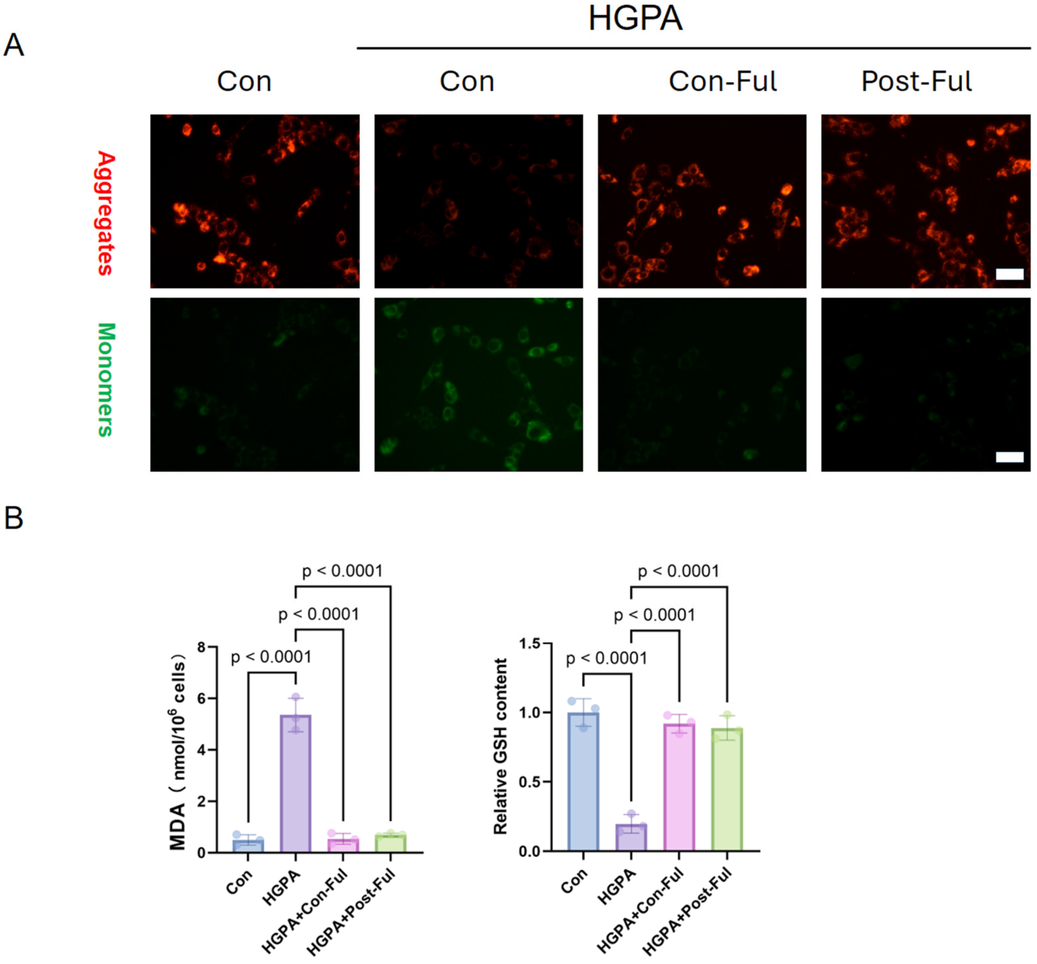

Figure S3: (A) JC-1 staining analysis of mitochondrial membrane potential. Representative fluorescence images of red (J-aggregates) and green (monomers) channels are shown for each treatment group. Scale bar = 50 μm. (B) Malondialdehyde (MDA) and glutathione (GSH) levels in C2C12 myoblasts under the indicated conditions. MDA was measured as a marker of lipid peroxidation, and total GSH content was determined as an index of cellular antioxidant capacity. Data are presented as the mean ± SD from three independent biological experiments (n = 3). For image-based analyses, three non-overlapping fields were analyzed per experiment

and averaged to generate one value for each biological replicate. Exact *p* values are shown in the corresponding figures.

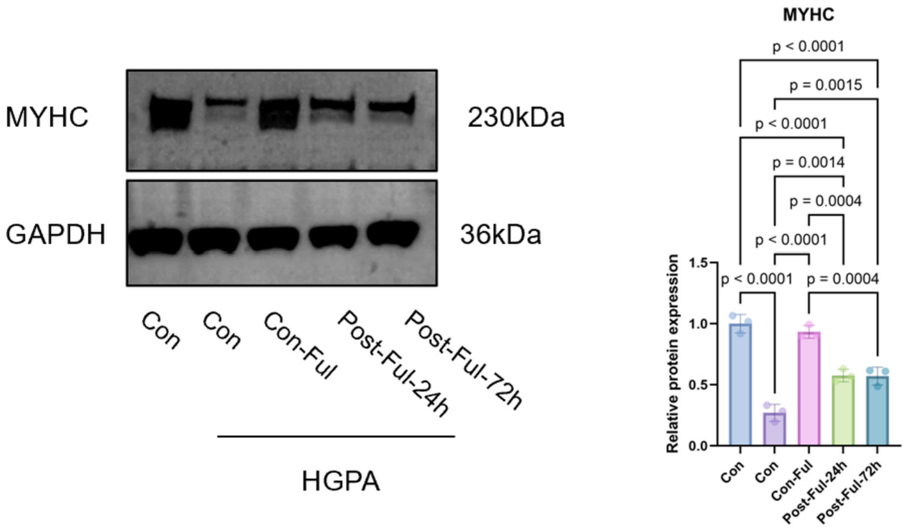

Figure S4: Representative western blot images and quantitative analysis of MYHC protein expression, with GAPDH used as the loading control. MYHC protein expression was assessed in parallel cultures after the 96-h treatment schedules followed by an additional 4 days in differentiation medium. Data are presented as the mean ± SD from three independent biological experiments (n = 3). Exact *p* values are shown in the corresponding figures.

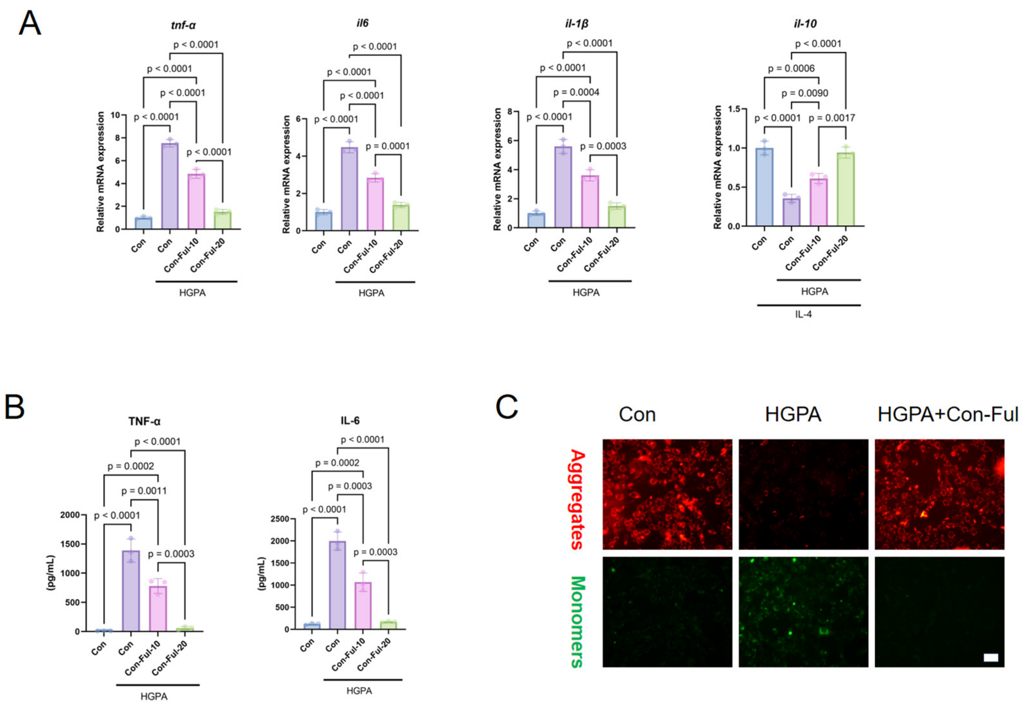

Figure S5: (A) Relative mRNA expression levels of *tnf-α*, *il6*, *il-1β*, and *il10* in RAW264.7 macrophages following the indicated treatments. (B) TNF-α and IL-6 concentrations were measured in aliquots of the same post-wash conditioned-medium preparations used to treat C2C12 myoblasts and were normalized to 1×10^7 viable donor macrophages. (C) JC-1 staining analysis of mitochondrial membrane potential. Representative fluorescence images of red (J-aggregates) and green (monomers) channels are shown for each treatment group. Scale bar = 50 μm. Data are presented as the mean ± SD from three independent biological experiments (n = 3). For image-based analyses, three non-overlapping fields were analyzed per experiment and averaged to generate one value for each biological replicate. Exact *p* values are shown in the corresponding figures.

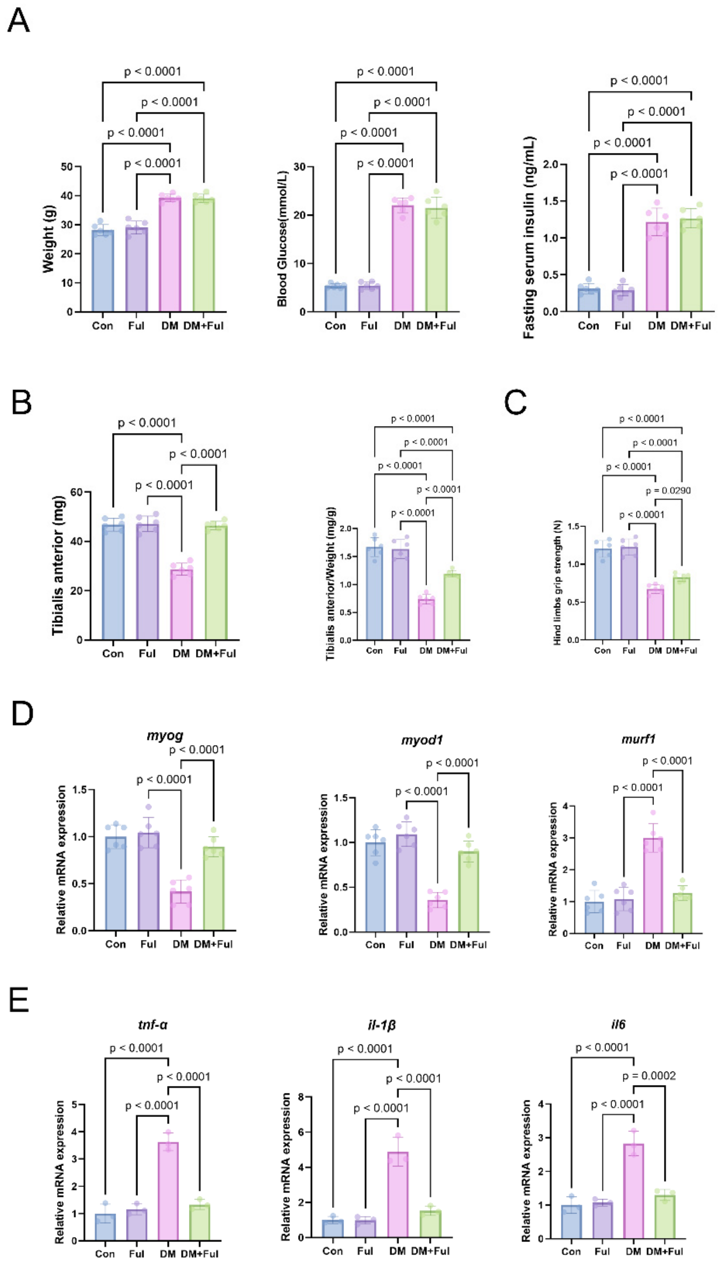

Figure S6: (A) Body weight, fasting blood glucose, and serum insulin levels at the end of the 12-week modeling period. (B) Absolute TA muscle weight and relative TA muscle weight normalized to body weight. (C) Hindlimb grip strength in the indicated groups. (D) Relative mRNA expression levels of the myogenesis-associated genes *myod1* and *myog* and the atrophy-associated gene *murf1* in TA muscle. (E) Relative mRNA expression levels of the inflammatory genes *tnf-α*, *il6*, and *il-1β* in whole-muscle tissue. Data are presented as the mean ± SD (n = 6 mice per group). Exact *p* values are shown in the corresponding figures. Con, non-diabetic vehicle-treated mice; Ful, non-diabetic mice receiving fullerol alone; DM, untreated diabetic mice; DM + Ful, diabetic mice receiving localized fullerol administration during model development.
